# Cyton2: A Model of Immune Cell Population Dynamics That Includes Familial Instructional Inheritance

**DOI:** 10.3389/fbinf.2021.723337

**Published:** 2021-10-26

**Authors:** HoChan Cheon, Andrey Kan, Giulio Prevedello, Simone C. Oostindie, Simon J. Dovedi, Edwin D. Hawkins, Julia M. Marchingo, Susanne Heinzel, Ken R. Duffy, Philip D. Hodgkin

**Affiliations:** ^1^ Hamilton Institute, Maynooth University, Maynooth, Ireland; ^2^ Immunology Division, the Walter and Eliza Hall Institute of Medical Research, Parkville, VIC, Australia; ^3^ Department of Medical Biology, the University of Melbourne, Parkville, VIC, Australia; ^4^ Oncology R&D, AstraZeneca, Cambridge, United Kingdom; ^5^ Division of Inflammation, the Walter and Eliza Hall Institute of Medical Research, Parkville, VIC, Australia; ^6^ Cell Signalling and Immunology Division, School of Life Sciences, University of Dundee, Dundee, United Kingdom

**Keywords:** immune response, population dynamics, mathematical model, familial correlations, proliferation

## Abstract

Lymphocytes are the central actors in adaptive immune responses. When challenged with antigen, a small number of B and T cells have a cognate receptor capable of recognising and responding to the insult. These cells proliferate, building an exponentially growing, differentiating clone army to fight off the threat, before ceasing to divide and dying over a period of weeks, leaving in their wake memory cells that are primed to rapidly respond to any repeated infection. Due to the non-linearity of lymphocyte population dynamics, mathematical models are needed to interrogate data from experimental studies. Due to lack of evidence to the contrary and appealing to arguments based on Occam’s Razor, in these models newly born progeny are typically assumed to behave independently of their predecessors. Recent experimental studies, however, challenge that assumption, making clear that there is substantial inheritance of timed fate changes from each cell by its offspring, calling for a revision to the existing mathematical modelling paradigms used for information extraction. By assessing long-term live-cell imaging of stimulated murine B and T cells *in vitro*, we distilled the key phenomena of these within-family inheritances and used them to develop a new mathematical model, Cyton2, that encapsulates them. We establish the model’s consistency with these newly observed fine-grained features. Two natural concerns for any model that includes familial correlations would be that it is overparameterised or computationally inefficient in data fitting, but neither is the case for Cyton2. We demonstrate Cyton2’s utility by challenging it with high-throughput flow cytometry data, which confirms the robustness of its parameter estimation as well as its ability to extract biological meaning from complex mixed stimulation experiments. Cyton2, therefore, offers an alternate mathematical model, one that is, more aligned to experimental observation, for drawing inferences on lymphocyte population dynamics.

## 1 Introduction

B and T lymphocytes are central contributors to the adaptive immune response. When exposed to a foreign pathogen with epitopes that are complementary to their B or T cell receptors, they respond by proliferating to create a clone army capable of recognising the threat. These cells differentiate into effector cells to fight the invasion, and into memory cells primed to fend off repeated insults. The population size of their response, the proportion of cells allocated to distinct differentiated effector types, the cytokines that they produce, and other key characteristics of the immune response are known to be heterogeneous but regulable (Kaech et al., 2002; Duffy et al., 2012; Buchholz et al., 2013; Gerlach et al., 2013). Variables that influence the outcome include the affinity of the receptor interaction and the provision of costimulatory signals from other cells (Marchingo et al., 2014). In the quest to better understand immune responses and therapeutic intervention, it remains an essential question to determine how signals are integrated to alter cell fate and how the cells process such information to yield diverse, yet appropriate outcomes. Answering this question requires an understanding of operational aspects of lymphocyte population dynamics, and the influence of signals on individual fates. When known, quantitative models and analytical techniques can be developed and used to monitor lymphocyte control under different conditions; they can recreate, and predict outcomes for complex situations (Duffy et al., 2012; Hodgkin, 2018).

Much of the understanding regarding lymphocyte population dynamics has come from assessing *in vitro* experiments. When isolated *ex vivo*, B and T cells are small, non-dividing, resting cells that die after a period of time if placed unstimulated into culture. The provision of activating signals leads to changes that reprogramme survival times and initiate cell division in quantitative manner (Gett and Hodgkin, 2000; Hawkins et al., 2007). After an initial period of intense transcriptional changes and cellular programming, activated cells initiate and undergo division repeatedly, before their offspring return to a non-dividing, quiescent state followed ultimately by death if no further signals, such as from cytokines, are received. Thus mathematical models for immune dynamics must have features that match biological processes and allow the alteration of division times, the number of cell divisions, the likelihood of cell death, and rules for how these parameters are altered by changes in signalling conditions.

Advances in experimental technologies have provided detailed data on lymphocyte population dynamics that have informed modelling frameworks. A key development came in 1994 with the discovery that cell divisions could be followed and enumerated by flow cytometry with fluorescent dye carboxyfluorescein diacetate succinimidyl ester (CFSE) (Lyons and Parish, 1994), with subsequent developments deriving distinct colours (Quah and Parish, 2012) including CellTrace™ Violet (CTV). After a short period of culture with these dyes cells become intensely fluorescent and measurable by flow cytometry. On division, their offspring inherit half their parent’s dye and so fluoresce with half their intensity. That methodology allows up to eight distinct generations to be measurable within a single culture by flow cytometry before fluorescence falls to a level indistinguishable from background. Data from CFSE and CTV experiments informed, for example, the mathematical models reported in Gett and Hodgkin (2000), Boer and Perelson (2005), Ganusov et al. (2005), Asquith et al. (2006), Hawkins et al. (2007), Luzyanina et al. (2007), Subramanian et al. (2008), Duffy and Subramanian (2009), Hyrien and Zand (2008), Zilman et al. (2010), Banks et al. (2011), Miao et al. (2011), Banks et al. (2012), Shokhirev and Hoffmann (2013), Mazzocco et al. (2017). Many of these models either ignore cell survival or assume that it is a fixed feature that is, independent of the age of cells. Most of these models also assume age independent division times to make stochastic systems Markovian or consider only the evolution of the average system, expressed as ordinary differential equations.

In contrast, directly performing novel experiments for the goal of mathematical model design, Hawkins et al. (2007) measured survival over time and concluded cell age was important to their fate. They also extended earlier work of Gett and Hodgkin (2000) that demonstrated that division and death times could be regulated independently within the same cell. Based on those data, they proposed a model where cell age and stochastic operations govern fate outcomes. Their Cyton model of the cell was named for the putative molecular machinery creating regulable timers for division and death.

In the Cyton model, division and death times are heterogeneous in the cell population and so modelled by random variables whose operation appears independent. Within each cell, the two timers are in competition, where whichever one completes its operation first determines the fate of the cell. This model structure gives rise to the prediction of distinctive correlations that are observed in data (Duffy and Hodgkin, 2012). In the absence of detailed information on individual cells and their offspring, the Cyton model assumed that timers were independently reset at each generation. To complete the Cyton model, an additional component was introduced: the number of divisions cells underwent before cessation of expansion and their return to quiescence. This parameter, termed division destiny (DD), was described by a probability of continuing motivation to divide after each cell division.

Thus, in the Cyton model a cell would divide rapidly for a period when division times outcompeted death times. The fate of a cell that stops dividing by triggering division destiny is then solely governed by its final death time. By adjusting the probability distributions of division, death and destiny, the model recreated typical immune cell population dynamics without further *ad hoc* assumptions (Hawkins et al., 2007; Subramanian et al., 2008; Lee et al., 2009; Wellard et al., 2011). After its development, the Cyton model was successfully used as a tool in important studies that extracted information on key features controlling immune dynamics (Hawkins et al., 2013; Shokhirev and Hoffmann, 2013; Marchingo et al., 2014; Shokhirev et al., 2015; Mitchell et al., 2018). Some of the assumptions on which the Cyton model was based were unobserved facets, and needed further experimental confirmation for their suitability. In particular, questions of familial correlation needed to be addressed by time-lapse microscopy and other, similarly capable, methods.

Stimulated lymphocytes typically aggregate, adhering together, making individual cell tracking by microscopy difficult or impossible. However, Hawkins et al. (2009) noted that B cells stimulated by the Toll-like receptor agonist CpG DNA exhibited the population dynamics typical of standard immune responses, but remained separated and individually identifiable (Hawkins et al., 2009). Using microscopy, the authors tracked over 180 individual family trees enabling statistical features such as dependencies to be assessed. Strikingly, it became apparent that division and death times of siblings were highly correlated. Further, division destiny, the number of divisions cells undergo before returning to quiescence, was a strongly familial feature (Hawkins et al., 2009). This conclusion, which ran contrary to assumptions underlying all previous mathematical models, was examined and further extended in subsequent studies Duffy and Subramanian (2009); Markham et al. (2010); Wellard et al. (2010); Duffy et al. (2012); Dowling et al. (2014); Shokhirev et al. (2015); Mitchell et al. (2018). In a parallel development, a division dye multiplex method, which provides less lineage information than live cell imaging, but has higher throughput for identifying families, was developed (Marchingo et al., 2016; Horton et al., 2018). When used with antigen stimulated CD8^+^ T cells, similar familial features to those observed directly for B cells were reported.

In addition to those population dynamics studies, the proto-oncogene Myc was identified as a molecular correlate that explained one important aspect of familial sharing of information. Results in Heinzel et al. (2017) established that in B and T cells Myc levels increase in response to mitogenic stimuli, and, so long as levels are sustained above a critical threshold, Myc acts as a license for cells to divide. Over the course of the response Myc levels then fall, and once they drop below the threshold, these cells lose their motivation for further division and re-enter quiescence. Crucially, that experimental work established that the time between cell divisions was uncoupled from the Myc level. Further, importantly, Myc levels altered over time, diminishing late in culture, but the kinetics of change were transmitted to offspring without being affected by mitosis. Taken together, these results indicate that the control of division destiny should be viewed as being timed, rather than counted by cell division (Heinzel et al., 2017). The familial inheritance of division destiny was consistent with the high correlations in fate within clonal families that were reported for both B and T cells (Hawkins et al., 2009; Duffy et al., 2012; Marchingo et al., 2016; Horton et al., 2018; Zhou et al., 2018). Heinzel et al. (2017) also reported evidence that time to death under these conditions was also programmed early in the stimulated cell and passed to descendants without being altered in a manner analogous to the transmission of the division destiny times. As a result, the fate of whole family members can be highly concordant while allowing significant variation of the times between families from an otherwise homogeneous cell population.

Collectively, these findings suggest alterations to current model paradigms are necessary. While the Cyton model was correct in its assessment of competing timers, assigning them to families rather than individual cells is more consistent with these data. Here, we propose and develop a new Cyton model where familial inheritance of times for destiny and survival fates are included. We examine datasets from time-lapse microscopy of B and CD8^+^ T cell families, and interrogate these data to investigate consistency with timed outcomes. We measure correlations in the likelihood of each alternative fate and determine a suitable class of parametric distributions for their description. The proposed model is constructed such that identifiability is improved while computational model fitting burden over the earlier Cyton model is not increased. We use the model to interrogate CTV stained datasets obtained using flow cytometry, illustrating its utility and efficacy when used with both B and T cells.

## 2 Results

### 2.1 Cyton2 Model Structure

**FIGURE 1:**
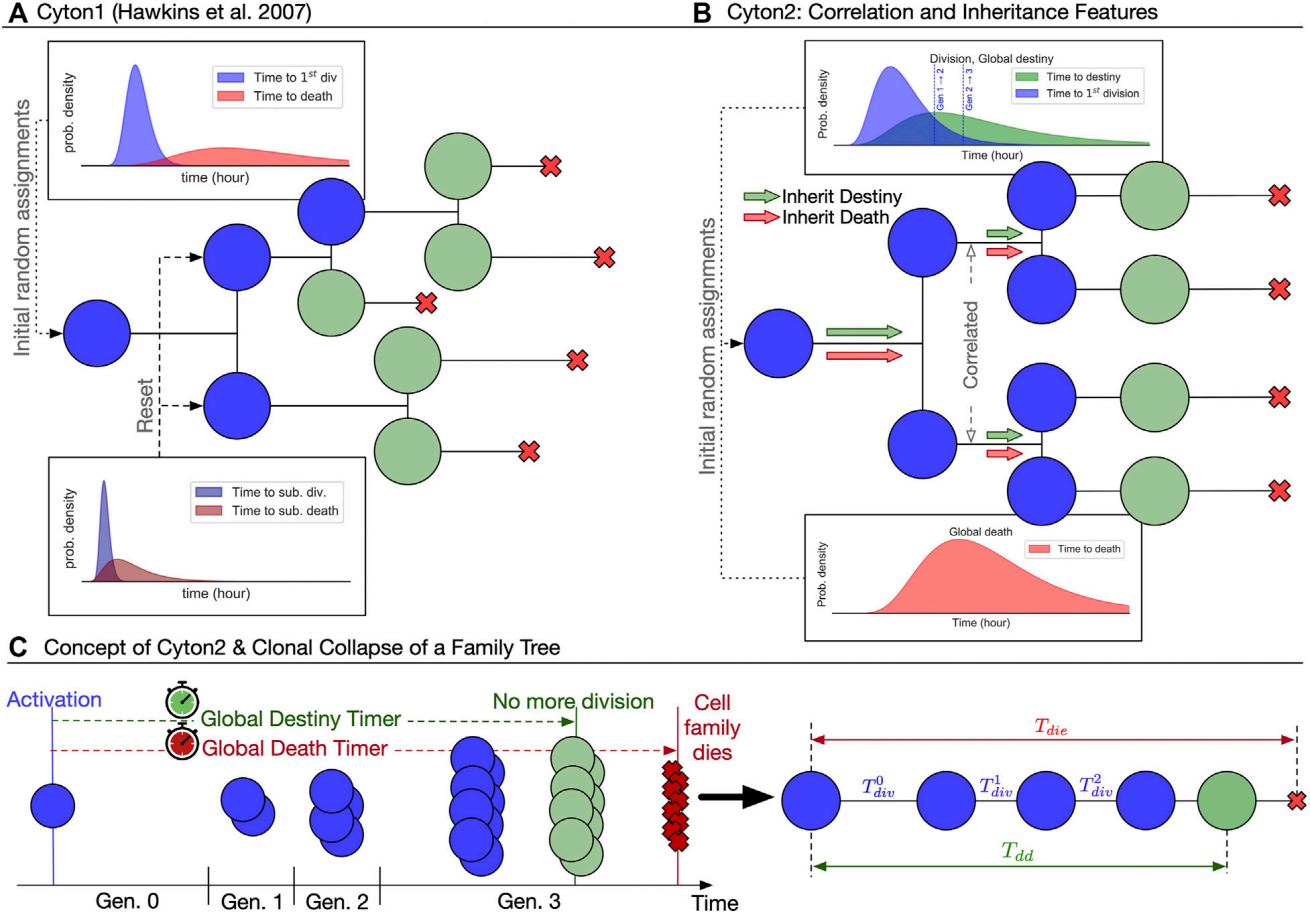
Overview of the two Cyton models. **(A)** The original Cyton model (Hawkins et al., 2007) where stochastic times to divide and to die are chosen independently after each cell division. Cells cease their motivation to divide based on division-counting mechanism. **(B)** The Cyton2 model incorporates significant correlation in division times between siblings, as well as familial inheritance of death and division destiny times. **(C)** A consequence of the correlation and inheritance is that the resulting family trees are heterogeneous, but highly concordant. By exploiting this property, a family tree can be summarised by substituting the average values of its times and fate at each generation. An example of clonally collapsed family tree and its key variables is shown.

Recent experimental findings suggest three important modifications to the original Cyton model for stimulus-induced proliferation bursts (Figure 1A): 1) division destiny should be converted to a division-agnostic, family-based timed mechanism, replacing the original generation counter; 2) both division destiny and death times should be programmed early after each lymphocyte’s activation and applied globally to the ancestor’s offspring; and 3) family members of the same generation should have essentially the same division time. As has been observed experimentally (Hawkins et al., 2009; Marchingo et al., 2016; Horton et al., 2018; Mitchell et al., 2018), the resulting family trees of activated lymphocytes derived from a single founder cell, and hence clones, according to Cyton2 rules are largely regular (Figure 1B). Thus we posit the new Cyton2 stochastic model using sets of random variables that correspond to a global death timer, a global destiny timer, and division-time machinery (Figure 1C).

The development of each family tree in Cyton2 is fully described by a collection of independent, non-negative, real-valued random variables: $\left(T_{div}^{0},{\left\{T_{div}^{k}\right\}}_{k\geq 1},T_{dd},T_{die}\right)$ . Three of these describe times from the addition of stimulus: the time to first division $T_{div}^{0}$ ; the time to familial division destiny *T*_*dd*_, encapsulating the licence to divide period; and the time to familial death *T*_*die*_. The last set of random variables, ${\left\{T_{div}^{k}\right\}}_{k\geq 1}$ are the times from each mitosis to the next, should it complete before division destiny or death occurs. From these random variables, a family tree is created according to the following rules:

• Founding cells that give rise to familial clones are initially quiescent, unrelated and autonomous.
• All cells in the family die at *T*_*die*_.
• The family proliferates until   min (*T*_*die*_, *T*_*dd*_).
• At time *t* < min (*T*_*die*_, *T*_*dd*_), cells in the family are in generation $G\left(t\right)=\max\left\{g:\sum_{k=0}^{g}T_{div}^{k}<t\right\}$ .

To properly assess the appropriateness of the Cyton2 as a fine-grained description required time-lapse microscopy data. To that end we re-analysed previously published B cell data sets as well as new, primary CD8^+^ T cell datasets.

### 2.2 Time-Lapse Microscopy of B and T Cell Families

**FIGURE 2:**
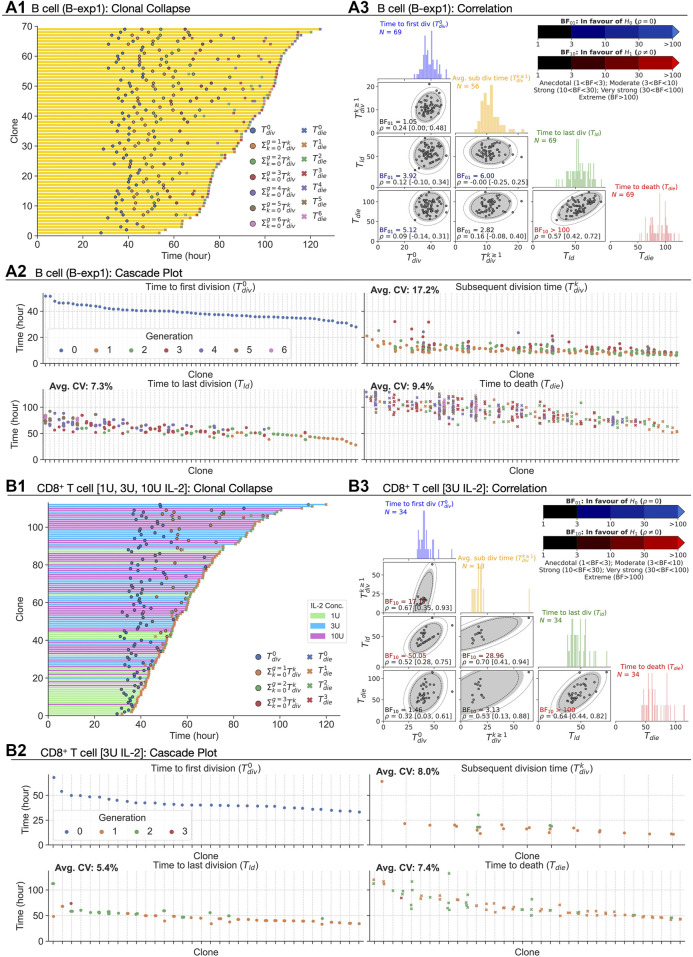
Extracting times to fates from CpG-stimulated B cells and CD8^+^ T cells in the presence of 1, 3, or 10 U IL-2. **(A1, B1)** Clonally collapsed family trees of B and T cells respectively. **(A2, B2)** Rank ordered times to events of families. **(A3, B3)** Correlation coefficient (*ρ*) estimated using bivariate normal distribution with 95% credible interval is reported for each pair. 90, 95 and 99% density regions are plotted over the data. For null, H_0_: *ρ* = 0, and alternative, H_1_: *ρ* ≠ 0, hypotheses, Bayes Factors (BF_01_ = 1/BF_10_) were calculated. If the data is more probable under H_0_, then it is BF_01_ times more favoured than H_1_ (blue-scale), and vice versa (red-scale). Distributions of the times are collated into 1 h time intervals and shown in the diagonal panels.

For B cells, we revisited two datasets for CpG-stimulated B cells published in Hawkins et al. (2009) consisting of 108 clones (B-exp1) and 88 clones (B-exp2), respectively. These datasets had not been analysed for timed global features but had revealed strong familial correlations previously (Duffy and Subramanian, 2009; Hawkins et al., 2009; Markham et al., 2010; Wellard et al., 2010). Thus, to explore familial features we first transformed the data for each family, collapsing the tree into average features (see Methods; Supplementary Table S1, Supplementary Figures S1, S2 for raw data). This process is illustrated in Figure 1C and was applied to each B cell family as shown in Figure 2A1. Measurements corresponding to key Cyton2 variables are further illustrated in the cascade plots Figure 2A2 with the exception of the time to division destiny (*T*_*dd*_) as it cannot be identified directly in data. Instead, the time to last division (*T*_*ld*_), which is necessarily a lower bound, was used as a proxy for it. These data reconfirmed the well-established understanding that times to first division, ≈ 40 h, are substantially longer than times to subsequent divisions. These data also confirmed the relatively consistent subsequent division times (≈10 h) and the strong correlation times between progeny cells within a given generation in each family.

Using these measurements, we evaluated the discrepancy between Cyton2’s approximation of perfect within-family correlation in subsequent division time ( $T_{div}^{k}$ ), time to last division (*T*_*ld*_), and time to death (*T*_*die*_). We calculated coefficient of variation (CV) per clone, and evaluated the average CV for each variable. For $T_{div}^{k}$ , *T*_*ld*_ and *T*_*die*_, we identified 17.2, 7.3, and 9.4%, respectively, as average CVs for B-exp1. Similar results were found for B-exp2 (see Supplementary Figure S3A1). This signifies low variation around the mean times to fates within families, and is consistent with previously reported synchronous behaviour. We then questioned the independence of the variables operating at the clone level using information from the collapsed clones. Here, for statistical purposes, we extracted the time to first division, average subsequent division time ( $T_{div}^{k\geq 1}$ ), average time to last division, and average time to death, as the four key variables per clone. For every pair, the correlation coefficient (*ρ*) and its 95% credible interval were determined using a Bayesian approach. For these data, the Bayes Factor (BF) for competing hypotheses (H_0_: *ρ* = 0 and H_1_: *ρ* ≠ 0) were calculated (see Methods) (Figure 2A3) and tabulated in Table 1. With the exception of the pair (*T*_*ld*_, *T*_*die*_), CpG-stimulated B cells showed little to no correlation between any pair of variables, with H_0_ being favoured. While at first glance the exception may appear suggestive of shared regulation, another explanation is possible, which is examined in the next section.

**TABLE 1:** Bayesian independence test of the times to fates extracted from B and CD8^+^ T cell filming datasets. For each pair of the times to fates, the correlation coefficient was estimated with 95% credible interval using bivariate normal distribution and Bayes Factor (BF) was calculated. Given two hypotheses (H_0_: *ρ* = 0 and H_1_: *ρ* ≠ 0), if the data is more probable under H_0_, then it is BF_01_ times more favoured than H_1_, otherwise H_1_ is BF_10_ times more favoured than H_0_.

Cell type	Stim	Number of clones (*N*) and bayes Factor^*a*^ (BF_01_ = 1/BF_10_) and correlation coefficient [*ρ* (CI)]					
		( $T_{div}^{0},T_{div}^{k\geq 1}$ )	( $T_{div}^{0},T_{ld}$ )	( $T_{div}^{0},T_{die}$ )	( $T_{div}^{k\geq 1},T_{ld}$ )	( $T_{div}^{k\geq 1},T_{die}$ )	(*T*_*ld*_, *T*_*die*_)
B (B-exp1)	CpG	*N* = 56	*N* = 69	*N* = 69	*N* = 56	*N* = 56	*N* = 69
		BF_01_ = 1.05	BF_01_ = 3.92	BF_01_ = 5.12	BF_01_ = 6.00	BF_01_ = 2.82	BF_10_ > 100
		0.24 [0.01, 0.48]	0.12 [−0.10, 0.35]	0.09 [−0.14, 0.31]	0.0 [−0.25, 0.25]	0.16 [−0.08, 0.40]	0.57 [0.42, 0.72]
CD8^+^ T	1U IL-2	*N* = 4	*N* = 28	*N* = 28	*N* = 4	*N* = 4	*N* = 28
		BF_01_ = 1.70	BF_10_ = 39.09	BF_01_ = 2.53	BF_01_ = 1.53	BF_01_ = 1.43	BF_10_ > 100
		−0.01 [−0.86, 0.85]	0.55 [0.29, 0.78]	0.19 [−0.15, 0.53]	−0.15 [−0.96, 0.71]	0.20 [−0.66, 0.98]	0.59 [0.35, 0.81]
	3U IL-2	*N* = 13	*N* = 34	*N* = 34	*N* = 13	*N* = 13	*N* = 34
		BF_10_ = 17.17	BF_10_ = 50.05	BF_10_ = 1.46	BF_10_ = 28.96	BF_10_ = 3.13	BF_10_ > 100
		0.67 [0.36, 0.93]	0.52 [0.28, 0.75]	0.32 [0.03, 0.61]	0.70 [0.41, 0.94]	0.52 [0.12, 0.87]	0.64 [0.44, 0.82]
	10U IL-2	*N* = 16	*N* = 50	*N* = 50	*N* = 16	*N* = 16	*N* = 50
		BF_10_ = 7.55	BF_10_ > 100	BF_10_ = 15.39	BF_10_ > 100	BF_10_ = 1.56	BF_10_ > 100
		0.57 [0.23, 0.86]	0.58 [0.41, 0.75]	0.40 [0.18, 0.62]	0.76 [0.55, 0.94]	0.42 [0.02, 0.79]	0.63 [0.46, 0.78]

^*a*^BF Interpretation: Anecdotal (1 < BF ≤ 3); Moderate (3 < BF ≤ 10); Strong (10 < BF ≤ 30); Very strong (30 < BF ≤ 100); Extreme (BF > 100).

Extending the analysis to T cells, we also interrogated three primary data sets of time-lapse microscopy of murine CD8^+^ T cells not previously published. In each dataset, TCR transgenic OT-I CD8^+^ T cells specific for the SIINFEKL (N4) peptide from the chicken ovalbumin protein (Hogquist et al., 1994) were first stimulated with *α*CD3 or cognate peptide N4 along with a range of costimulatory signals and strengths for 24 h. In the first dataset (i) the cells were stimulated with *α*CD3 and co-incubated with 1, 3, or 10 U/mL of the T-cell growth factor IL-2. IL-2 level was buffered by neutralising endogenously produced IL-2 with blocking antibody S4B6, and adding human IL-2 at the nominated concentration (Deenick et al., 2003). By combining datasets obtained from two independent repeats, 109, 90, and 163 clones were recorded. In (ii), the combination of N4, *α*CD28, and IL-2 were used (T-exp1); and, in experiment (iii) the combination of N4, *α*CD28 and IL-12 (T-exp2). Details for live imaging and data extraction are given in Methods. In Figure 2B, results from CD8^+^ T cell dataset (i) are aggregated and analysed as for B cells. Similar to B cells, we observed longer times to first division (≈40 h) than the subsequent division times (≈18 h) for 1, 3, and 10 U of IL-2. Also, the spread of the times within a family show similar or lower average CVs than that of B cells (Figure 2B2 for 3 U; see Supplementary Figures S3B1,C1 for 1 and 10 U). We applied the same calculation to (ii) and (iii) datasets and reached the same conclusions (see Supplementary Figures S4A,B for T-exp1 and T-exp2). Taken together, we conclude CD8^+^ T cells exhibit synchronous fates, similar to observations from B cells. However, in contrast to B cells, moderate to strong correlation coefficients were observed (Figure 2B3). These were further supported by BF calculations, which show strong evidence in favour of H_1_ (Table 1). We noticed the same results for T-exp1 and T-exp2 datasets (see Supplementary Table S2).

At face value, as with the pair (*T*_*ld*_, *T*_*die*_) for B cells, these data are suggestive of a lack of stochastic independence between underlying timers. An alternate explanation is, however, possible and we next sought to challenge it.

### 2.3 Induced Dependency Through Right Censoring of Timers

Informed by earlier data, in constructing Cyton2 we assumed that $\left(T_{div}^{0},{\left\{T_{div}^{k}\right\}}_{k\geq 1},T_{dd},T_{die}\right)$ were independent random variables describing times to familial events. In the data, however, not all of them are observable due to a phenomenon called right-censoring. In particular:

• If $T_{div}^{0}$ or $T_{div}^{k}$ is greater than either of *T*_*dd*_ or *T*_*die*_, it is not observed in the data.
• If *T*_*dd*_ is greater than *T*_*die*_, it is not observed in the data.

Even if the underlying random variables are independent, right-censoring necessarily induces correlation in times observed in data (Duffy et al., 2012; Duffy and Hodgkin, 2012) where the greater the competition in these times, the stronger the observed correlation. While these earlier demonstrations of censorship-induced correlations were seen within one generation, we explored the possibility that heritable fates times across multiple generations could also lead to a similar effects.

**FIGURE 3:**
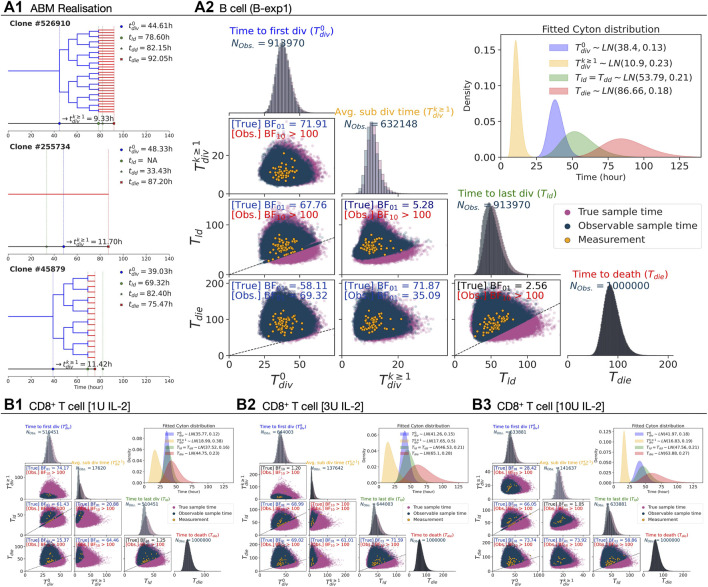
Simulation under the independence assumption. 10^6^ Cyton2 families were simulated given fitted lognormal distributions of $T_{div}^{0}$ , $T_{div}^{k\geq 1}$ , *T*_*ld*_, *T*_*die*_ from respective filming datasets. **(A1)** Three example ABM families parameterised as CpG-stimulated B cells: dividing (blue solid-line) and dying (red solid-line) states. The realisations of $T_{div}^{0}$ (•), $T_{div}^{k\geq 1}$ ( $t_{div}^{k\geq 1}$ ), *T*_*ld*_ (•), *T*_*dd*_ (⋆) and *T*_*die*_ (×) are annotated on a collapsed line. As a feature of inheritance, the progeny cells double in number synchronously whenever division occurs, and likewise, they reach destiny and death at the same time. **(A2, B1–B3)** For all simulated families, each variable was randomly sampled from the fitted Cyton distribution (inset), and the samples are labeled as true sample time (purple dot). Their corresponding observable sample times (blue dot) are shown along with the data points (orange dot) from the filming datasets. Distributions of the sampled true and observable times of each variable are shown in the diagonal panels. The observable and unobservable regions are separated by upper and lower sections of *y* = *x* line (dashed-line), respectively. The Bayes factors are reported for the true and observable pairs.

In Section 2.2, most of the variable pairs for B cell families were reported to be more probable under the no-correlation hypothesis, while for the CD8^+^ T cell families we found mixed results. The key difference between the two datasets is the depth of the trees: many of the B cell families had divided six times, whereas the CD8^+^ T cell families had divided at most three times (Figures 2A2,B2). This suggests the possibility that more of the variables are rendered unobserved for the CD8^+^ T cell families. To challenge that possible explanation, we simulated a Cyton2 process with an agent based model (ABM) (see Methods). As *T*_*dd*_ is not directly observable in data, we use *T*_*ld*_ as a proxy for it. As *T*_*dd*_ ≥ *T*_*ld*_, this approximation may lead to an increase in the level of induced censorship. Under the assumption that *T*_*ld*_ = *T*_*dd*_, each variable was independently sampled from respective lognormal distributions that were fit to the data (see Section 2.4). In Figure 3A1, three example realisations of family trees are shown for a parameterisation corresponding to CpG-stimulated B cells.

For B cells, with each point representing a single family, the underlying simulated variable values as well as those that would appear in the data due to the right-censorship described above are shown in Figure 3A2. By construction, the BFs for each pair in the underlying timers favour the null hypothesis (H_0_: *ρ* = 0). For the right-censored values that would be observed in practice, however, the BFs are consistent with the experimental data in favouring the alternative hypothesis (H_1_: *ρ* ≠ 0) for some pairs (Table 2). As the underlying *T*_*die*_ distribution is well separated from the $T_{div}^{0}$ distribution for these data, it is unlikely that death would censor the time to first division, hence, it explains why the absence of correlation in the observed data is favoured in that case. Similar results were found for B-exp2 (data not shown). We followed a similar protocol for the CD8^+^ T cell data where a higher degree of right-censorship occurs due to the underlying distributions having greater overlap (Figures 3B1–3). The BFs of all right-censored pairs were in favour of H_1_, indicating strong correlation between times to fates for CD8^+^ T cells are to be expected in the observed data as a result of high degree of right-censorship. Thus, despite the temporal-correlations observed in the data, right-censorship supports our assumption in the Cyton2 model that the underlying stochastic variables are independent. This statistical conclusion complements the experimental evidence for fate independence obtained by slowing division times and preventing cell death without altering other outcomes (Heinzel et al., 2017).

**TABLE 2:** Bayesian independence test of the times to fates from simulation. The test was performed with *N*_True_ = 10^6^ simulated families *via* Cyton2-like Agent-Based Model. Each family was assigned randomly sampled times (True), and corresponding observable (Obs.) times were recorded. Depending on the order of the true times to fates, the number of observable times (*N*_Obs._) may vary. For both true and observable times, Bayes Factor (BF) was calculated given null and alternative hypotheses (H_0_: *ρ* = 0 and H_1_: *ρ* ≠ 0). Here BF_01_ indicates the simulated data are more probable under H_0_, otherwise it is indicated by BF_10_.

Cell type	Stim	Percentage of number of observable times (*N*_Obs._/*N*_True_) and bayes Factor^*a*^ (BF_01_ = 1/BF_10_)											
		( $T_{div}^{0},T_{div}^{k\geq 1}$ )		( $T_{div}^{0},T_{ld}$ )		( $T_{div}^{0},T_{die}$ )		( $T_{div}^{k\geq 1},T_{ld}$ )		( $T_{div}^{k\geq 1},T_{die}$ )		(*T*_*ld*_, *T*_*die*_)	
		True	Obs	True	Obs	True	Obs	True	Obs	True	Obs	True	Obs
B (B-exp1)	CpG	BF_01_ = 71.91	63.2%	BF_01_ = 67.76	91.4%	BF_01_ = 58.11	91.4%	BF_01_ = 5.28	63.2%	BF_01_ = 71.87	63.2%	BF_01_ = 2.56	91.3%
			BF_10_ > 100		BF_10_ > 100		BF_01_ = 69.32		BF_10_ > 100		BF_01_ = 35.09		BF_10_ > 100
CD8^+^ T	1U IL-2	BF_01_ = 74.17	17.6%	BF_01_ = 61.43	51.0%	BF_01_ = 15.37	51.0%	BF_01_ = 20.88	17.6%	BF_01_ = 64.46	17.6%	BF_01_ = 1.25	51.0%
			BF_10_ > 100		BF_10_ > 100		BF_10_ > 100		BF_10_ > 100		BF_10_ > 100		BF_10_ > 100
	3U IL-2	BF_10_ = 1.20	13.8%	BF_01_ = 68.99	64.4%	BF_01_ = 69.02	64.4%	BF_10_ > 100	13.8%	BF_01_ = 61.01	13.8%	BF_01_ = 71.59	64.4%
			BF_10_ > 100		BF_10_ > 100		BF_01_ > 100		BF_10_ > 100		BF_10_ > 100		BF_10_ > 100
	10U IL-2	BF_01_ = 28.42	14.1%	BF_01_ = 66.05	63.2%	BF_01_ = 73.74	63.2%	BF_01_ = 1.05	14.1%	BF_01_ = 73.92	14.1%	BF_01_ = 58.86	63.2%
			BF_10_ > 100		BF_10_ > 100		BF_10_ > 100		BF_10_ > 100		BF_10_ > 100		BF_10_ > 100

^*a*^BF Interpretation: Anecdotal (1 < BF ≤ 3); Moderate (3 < BF ≤ 10); Strong (10 < BF ≤ 30); Very strong (30 < BF ≤ 100); Extreme (BF > 100).

### 2.4 Using Filming Data to Determine Appropriate Distribution Classes for the Timers

In order to fit the model to commonly available non-microscopy data where direct observation of times is not possible, it is necessary to determine appropriate parametric distribution classes that well-capture the structure of the timers. Probability distributions governing the times to first division and to death for B cell cultures have been reported to be well approximated by a right-skewed distribution such as Lognormal, Weibull, Gamma, or Beta (Hawkins et al., 2009). In particular, the time to first division is known to be better described by a Lognormal rather than other skewed distributions, whereas Gamma or Weibull distribution can be used to approximate the time to death distribution (Hawkins et al., 2007).

**FIGURE 4:**
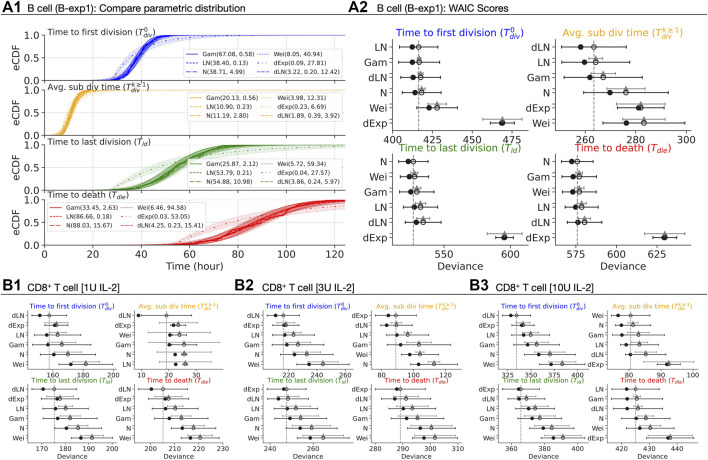
Best parametric distribution class. **(A1)** Empirical CDF of the measured times are overlaid with CDFs of Gamma, Lognormal, Normal, Weibull, delayed Exponential and delayed Lognormal distributions. 95% confidence bands are plotted by randomly drawing 10^4^ samples from respective posterior distributions. **(A2, B1–B3)** The in-sample deviance (dot), and WAIC scores (open-circle) with 1 standard deviation error bar are shown. The *y*-axis is sorted from lowest **(top)** to highest **(bottom)** WAIC score. The lower WAIC score indicates better descriptor of the data. Except the top-ranked one, the value of the difference of WAIC (grey triangle) between a candidate and the top-ranked are shown with 1 standard error of the difference.

For B cells, the empirical cumulative distribution function (eCDF) measured times overlaid with CDFs of four candidate distributions with 95% confidence bands in Figure 4A1. Each candidate is parameterised by: (i) (*α*_*G*_, *β*_*G*_) for Gamma; (ii) (*m*, *s*) for median and shape of Lognormal; (iii) (*μ*, *σ*) for mean and standard deviation for Normal; (*α*_*W*_, *β*_*W*_) for Weibull; (*λ*, *c*) rate and shift for delayed Exponential; and, (*m*_*d*_, *s*_*d*_, *c*) median, scale and shift for delayed Lognormal. Qualitatively, most of the candidates appear to be excellent descriptors for each of the measurements except for the delayed Exponential distribution for B cells. Here, we used the Widely Applicable Information Criterion (WAIC - see Methods) to quantitatively determine the best fit (Figure 4A2) (Watanabe and Opper, 2010). For $T_{div}^{0}$ in the B cell data, the Lognormal distribution was top-ranked (416.3) while delayed Exponential was least favoured (468.9, ΔSE = ±12.3). The delayed Lognormal was most preferred candidate for $T_{div}^{k\geq 1}$ (263.4), but the Lognormal was a close second (264.2, ΔSE = ±2.7). While the delayed Exponential was consistently worst fit for all measurements in B cells, the other five candidates well approximate *T*_*die*_ measurements as reported in previous studies. Interestingly, the Normal was favoured (526.9), or on par with the Weibull and Gamma, for *T*_*ld*_ as indicated by the standard error of the difference. The delayed Lognormal was second least preferred, however, the difference was relatively marginal compared to the Normal (534.3, ΔSE = ±4.9). We observed similar results in the repeat of B cell data (B-exp2), except for *T*_*die*_ where the Weibull provided the best fit (see Supplementary Figures S5A1–2).

For CD8^+^ T cells, we present the rank-ordered WAIC plot for all IL-2 concentrations in Figures 4B1–3 (see Supplementary Figures S5B1–3 for corresponding CDF plots). We observed either the delayed Lognormal or the delayed Exponential to be the best descriptor for all measurements with the exception of $T_{div}^{k\geq 1}$ in 10 U IL-2 in which Weibull was top-ranked (80.5), but only marginally so compared to the Normal (81.3, ΔSE = ±1.6), the Gamma (83.0, ΔSE = ±3.3) and the Lognormal (83.3, ΔSE = ±2.6). Note that the estimates of WAIC for $T_{div}^{k\geq 1}$ in 1 U IL-2 are unreliable as there are only four data points due to lack of progression of cell division in those conditions. We reached similar conclusions for both T-exp1 and T-exp2 datasets where the delayed Lognormal and the delayed Exponential were strongly and consistently preferred or was on par with other candidate distributions (see Supplementary Figure S6).

In summary, these data suggest that several parametric classes of distributions are well-suited as descriptors. We will, however, provide one example analysis of flow cytometry data where use of Gaussian distributions offer an interpretative advantage over the right-skewed distributions. Moreover, as time to subsequent division has little variability, when fitting fluorescence-activated cell sorting (FACS) data, we will use a reduced model that assumes it is an unknown constant that is, fit.

## 3 Application to FACS Data

While information from time-lapse microscopy has informed core elements of the Cyton2 model, in practice higher-throughput methodologies are typically employed in immunological investigation. In particular, it is common to have bulk experiments that start with a large number of initial cells that have been cultured with a division tracking dye and are then exposed to stimuli for a time-course of measurements by flow cytometry. Thus it is essential that any mathematical model can be fit to such data and extract biologically meaningful information from them. To that end, we derived expressions for the expected time-course per generation of the Cyton2 model and a least-squares fitting methodology, as described in Methods, for fitting to such data to challenge Cyton2’s applicability. We challenged the model with both B and CD8^+^ T cell datasets.

### 3.1 B Cell Data: Assessing Model Fits

We interrogated a primary dataset consisting of *in vitro* CpG-stimulated murine Bim^−/−^ B cells with cell numbers recorded in each generation via flow cytometry. Cells taken from this mouse strain are deficient in the pro-apoptotic molecule Bim (B-cell-lymphoma-2-like protein 11, or Bcl2l11). As a result, these cells survive longer in culture without impacting any other population dynamic feature (Turner et al., 2008). Here, we asked if a standard division tracking assay, which typically has three replicates at each of five or six harvested time points, provides sufficient information to well constrain model fits. To that end, the dataset that we used consists of nine replicates, collected at nine distinct time points.

**FIGURE 5:**
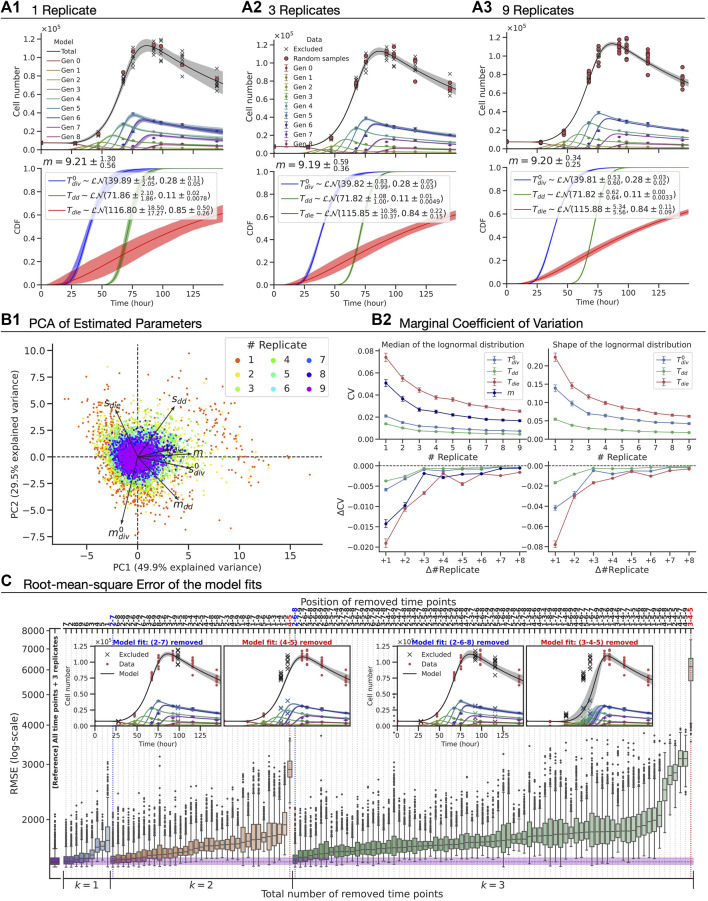
The precision of the parameter estimates and the accuracy of the model fit with CpG-stimulated Bim^−/−^ murine B cell FACS data. **(A1–A3)** The best-fit model **(top)**, which has seven fitted parameters (bottom): $T_{div}^{0}∼LN\left(m_{div}^{0},s_{div}^{0}\right),T_{dd}∼LN\left(m_{dd},s_{dd}\right),T_{die}∼LN\left(m_{die},s_{die}\right)$ and, subsequent division time, *m*. For a given replicate number, the model was fitted to 1,000 synthetic datasets, which were created by randomly sampling the original data with replacement per time point. **(B1)** From the sets of estimated parameter vectors, biplot of principle component analysis (PCA) result is shown. **(B2)** The marginal coefficients of variation (CV) was calculated with 95% confidence interval from bootstrapping. **(C)** The root-mean-square error (RMSE) was evaluated over all available data points after fitting the model to the synthetic datasets. The reference (purple) was obtained after fitting the model to datasets assuming only three replicates are available while maintaining all time points. Examples of the best (blue) and worst (red) fits are shown for 2 and 3 time-points being removed.

To assess the amount of data required to ensure a constrained model fit, we altered the amount of data used according to two scenarios: (i) varying the number of replicates sampled at all time points; and (ii) removing some of the time points while maintaining the number of replicates. In Figures 5A1–3, the best-fit model and the estimated parameters with 95% confidence intervals are shown (see Methods). Qualitatively, we observed that the confidence bands of the model fit get narrower around the mean as we increase the number of replicates, indicating an improvement in the model constraints, albeit with a law of diminishing returns. As estimated model parameters are coupled, we assessed their vector values using principle component analysis (PCA) (Figure 5B1). The PCA result signifies that the first two principle components explain 79% variability in the set and, furthermore, is suggestive that there is no notable correlation amongst components of the parameter vector. To assess the precision of the estimated parameters, we computed coefficients of variation for individual parameters as a function of the number of replicates (Figure 5B2). Again, a law of diminishing returns is observed with no significant benefit in precision of the estimates beyond three replicates. This suggests that the existing operational standard of three replicates offers a good balance between obtaining a precise estimate and managing experimental burden.

We turned our attention to evaluating the model accuracy as time points were removed while maintaining a fixed number of replicates (Figure 5C). For time-point removal, we imposed the following rules to avoid any ambiguity and ensure the feasibility of the model fits:

1) At least three time points must be retained.
2) Either the first or second time point must remain in order to provide an initial cell number for the model.

Given these rules, there are 366 cases to consider in total. For each case, we constructed 1,000 artificial datasets by randomly sampling three replicates with replacement per remaining time point, fitted the model assuming all random variables were log-normally distributed and then calculated the root-mean-square error (RMSE) of the model-fit from the original unaltered dataset with nine replicates at nine time points. In Figure 5C, we present rank ordered values of the RMSE when one, two or three time-points are removed (see Supplementary Figure S7 for greater than three). The RMSE of model fits using all time points with three replicates is also shown as a reference. Perhaps unsurprisingly, the results showed that capturing a measurement at the time at which cells are expanding is the most important information to be kept for the model accuracy. Intuitively, this would represent a regression of a non-linear curve in which data points around “inflection point” are missing while two ends points are present. Furthermore, we noticed that the first time point is generally more important than the later ones as RMSEs are higher if the first time point was removed. We found little to no difference in the RMSE compared to that of the reference when the positions of the removed time points are sparsely located. As an extreme example with six removed time-points, the model was capable of accurately fitting the data as long as there were three time points that correspond to the early (prior to first division), expansion and contraction phases (e.g., first, fourth and ninth time points, see Supplementary Figure S7B). Knowing in advance those three time points prior to an experiment is unrealistic, and so it represents a lower bound on the number of time-points needed. Removing more than six time-points, the model failed to fit due to the lack of information (results not shown). In summary, this analysis illustrates that Cyton2 is well constrained by data employing standard experimental protocols for following cell expansion by flow cytometry.

### 3.2 T Cell Data: Assessing Cyton2’s Ability to Draw Biologically Meaningful Inferences

To evaluate the utility of the model in drawing biologically useful inferences, we used it to reassess the non-linear population dynamics of experiments reported in Marchingo et al. (2014). That study established that CD8^+^ T cells integrated a range of distinct mitogenic stimuli *via* a simple, additive rule for the number of rounds of division they provoked. We questioned how the phenomenon could be understood in light of the new paradigm of familial concordance and global timers as realised in Cyton2.

This data was obtained from *in vitro* CTV-labeled OT-I/Bim^−/−^ CD8^+^ T cells stimulated with the peptide N4 and cultured with co-stimulatory antibodies CD27 (5 *μ*g/ml) and CD28 (2 *μ*g/ml), both alone and in combination. Cells were harvested at 27, 44, 52.5, 66.5, 69, 76.5, 90, 101, and 115.5 h after the stimulation with three replicates at each time point (Marchingo et al., 2014). Mirroring the deduction in the original paper, but expressing it in terms of timers, we sought to ask whether the contribution to division destiny of each co-stimulatory molecule in terms of time could be described by a simple additive process. As there is no simple closed form for the distribution of the sum of two independent lognormally distributed random variables, for this application we instead chose to fit Gaussian distributions. That is, assuming that ${T_{dd}}^{\text{N}4}$ is normally distributed with mean *μ*_N4_ and variance $\sigma_{\text{N}4}^{2}$ , i.e $N\left(\mu_{\text{N}4},\sigma_{\text{N}4}^{2}\right)$ , $T_{\text{d}d}^{\alpha CD27}$ is $N\left(\mu_{\alpha CD27},\sigma_{\alpha CD27}^{2}\right)$ , and $T_{\text{d}d}^{\alpha CD28}$ is $N\left(\mu_{\alpha CD28},\sigma_{\alpha CD28}^{2}\right)$ , if the contributions of *α*CD27 and *α*CD28 to division destiny time were problematically independent and additive, then we would expect that

$${T_{dd}}^{\alpha CD27+\alpha CD28}∼N\left(\mu_{\text{N}4}+\Delta \mu_{\alpha CD27}+\Delta \mu_{\alpha CD28},\sqrt{\sigma_{\text{N}4}^{2}+\Delta \sigma_{\alpha CD27}^{2}+\Delta \sigma_{\alpha CD28}^{2}}\right),$$ (1)

where Δ*μ*_*x*_ = *μ*_*x*_ − *μ*_N4_ and $\Delta \sigma_{x}^{2}=\sigma_{x}^{2}-\sigma_{\text{N}4}^{2}$ for *x* ∈ {*α*CD27, *α*CD28}.

**FIGURE 6:**
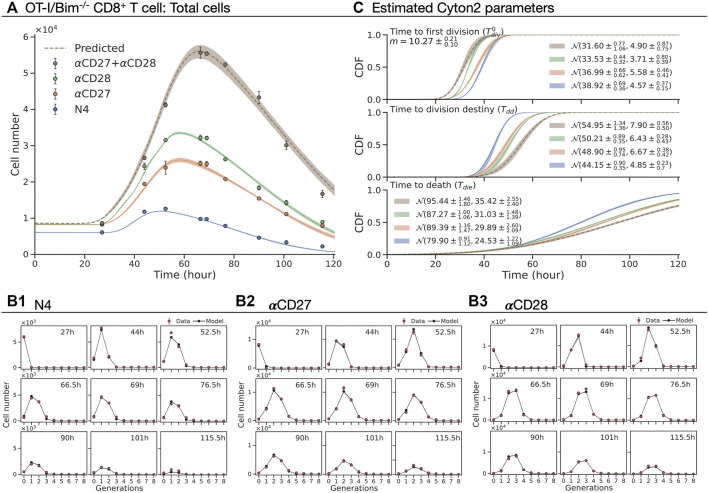
Fitting the Cyton2 model to OT-I/Bim^−/−^ CD8^+^ T cell FACS data (Marchingo et al., 2014). The cells were stimulated with N4 as basis for all other conditions. **(A)** Harvested total cell numbers (dot: mean ± SEM) overlaid with the model extrapolation and 95% confidence band from bootstrapping. **(B1–B3)** Live cells per generation and the model extrapolation at harvested time points. **(C)** 19 jointly fitted parameters ( $T_{div}^{0},T_{dd},T_{die}$ for each of N4, *α*CD27 and *α*CD28; a shared subsequent division time, *m*) and their 95% confidence intervals. The fitted and predicted values of mean and standard deviation are labelled in the legend for normally distributed random variables.

In Figure 6A, we present the total number of cells and the best-fit model with a 95% confidence band around the estimate from the original data. The model was simultaneously fitted to N4, *α*CD27, and *α*CD28 datasets with a shared subsequent division time (see Methods) (Figures 6B1–3), omitting the *α*CD27 plus *α*CD28 dataset for out of sample testing. The estimated *m* and cumulative distribution function (CDF) of $T_{div}^{0}$ , *T*_*dd*_ and *T*_*die*_ are shown in Figure 6C. In comparison to N4 alone, the addition of *α*CD27 and *α*CD28 extends both means of *T*_*dd*_ (≈15%) and *T*_*die*_ (≈10%). Also, we identified *α*CD28 reduces mean of $T_{div}^{0}$ (13.3%) while *α*CD27 has minimal impact. Collectively, the compounding effect of these changes results in larger expansion of cell numbers by allowing cells to enter the first division early and to reach destiny and death at later times. Given the parameter estimates for N4, *α*CD27 and *α*CD28, we predicted the number of cells for their combined effect by calculating the $T_{div}^{0},T_{dd}$ and *T*_*die*_ according to Eq. 1. Strikingly, this successfully recreated the expansion kinetics of OT-I/Bim^−/−^ CD8^+^ T cells in the presence of both *α*CD27 and *α*CD28 (Figure 6A), supporting the signal integration as a linear sum in a time domain of three dimensions and consistent with the independence of the timers. Additionally, we recapitulated the additive nature of mean division number presented in Marchingo et al. (2014) using ABM given the fitted and predicted parameter estimates (see Supplementary Figure S8). These results illustrate the merit of Cyton2 in uncovering how simple operations can underlie highly non-linear population dynamics.

## 4 Discussion

The vast majority of published mathematical models of lymphocyte population dynamics employed assume that a newly born cell’s fate is independent of its family’s history (Smith and Martin, 1973; Nordon et al., 1999; Revy et al., 2001; Ganusov et al., 2005; Yates et al., 2007; Lee et al., 2009; Banks et al., 2012; Hasenauer et al., 2012; Mazzocco et al., 2017), with a few notable exceptions (Hyrien et al., 2010; Wellard et al., 2010; Zilman et al., 2010; Shokhirev et al., 2015; Yates et al., 2017). These assumptions are adopted, not because they are consistent with experimental data from, for example, filming, FACS and lineage tracing, but for reasons of parsimony, model identifiability and computational ease of fitting (Dowling et al., 2005; Boer et al., 2006). In this work, we have presented a variant of the original Cyton model that encapsulates features of inheritance and correlation structure of cell fates. This was achieved by introducing new random variables that describe the time to division destiny of a family and a global death time, which describes a single death time for all members in a family tree. Similar to the Cyton model, this variant offers a general tool for analysing lymphocyte proliferation and survival, including from the data obtained from CFSE/CTV-labeled division tracking assays. Despite concerns that the inclusion of familial effects might result in a model with too many parameters or one that is, hard to fit, neither proves to be the case, making the model suitable for general use.

The analysis of the B and CD8^+^ T cell filming data allowed direct tests on the model’s assumptions of independent timers. Additionally, it enabled us to assess the suitability of classes of parametric distributions of random variables of the Cyton2 model, which is necessary for when the model is used with commonly available non time-lapse microscopy data. As there is no theoretical reason to favour one distribution class over another and several classes provide good fits to data, for most of our fitting examples we adopted the lognormal distribution class.

To fit Cyton2 to ubiquitous FACS data, we derived an expression for the mean population dynamics with one constant and three sets of distribution parameters. The random variables represent times to first division, to division destiny and to death, and the constant captures the subsequent division time. In the present work, the model was designed for cell populations exposed to newly available stimuli. In future work we will consider the inclusion of repeated challenges and continuous feedback mechanisms as occur, for example, with autocrine signalling *via* IL-2. Alterations to the model that allow the inclusion of ongoing signalling, as likely occurs when fighting replicating pathogens such as viruses or bacteria, will be the subject of future development. Moreover, the cell population model considered here does not include differentiation or other developments that would create asymmetries through altered division times, destiny or survival. Such alternative fates can arise from analogous competing outcomes promoting differentiation (Duffy et al., 2012), and we anticipate that the basic Cyton2 framework introduced here will be expanded to encompass additional fates as experimental information for the control of differentiation is acquired.

We provided two illustrative uses of the model through the analysis of FACS data from stimulated B and CD8^+^ T cell cultures. The first one provides quantitative support for the standard experiment design of triplicates per time-point, but also elucidates the importance of including time-points around the initial expansion and final contraction of the population. The second example revisits the work of Marchingo et al. (2014) addressing the question of signal integration by T cells. While the original study was informed based on modelling paradigms available at the time, reconsideration of it terms of family-based timers draws similar conclusions on additivity, but with a distinct temporal understanding that will influence all subsequent studies. Taken together these results suggest Cyton2 will prove to be a powerful tool in the quantitative assessment of immune responses. We anticipate the model will be useful in evaluating signal processing and genetic differences in both murine and human T and B cells, and will facilitate comparisons between healthy and unwell individuals.

The model is informed by, and the worked examples are for, data from *in vitro* experiments where stimulation is provided to a group of T or B cells, and the resulting proliferation occurs in a burst that can be followed by division tracking dyes or direct filming. Those population dynamics follows the pattern of an exponential rise, a period of division cessation, and then of cell loss that characterises immune responses *in vivo* (Veiga-Fernandes et al., 2000; Costa Del Amo et al., 2020). As such, as with the original Cyton model (Hawkins et al., 2007; Subramanian et al., 2008; Marchingo et al., 2014), Cyton2 can be successfully fit to *in vivo* data (data not shown). We note, however, that for most *in vivo* data stochastic models offer no advantage over models, such as those based on ODEs, that assume transitions to distinct phases and require fewer parameters (Boer and Perelson, 2005; Ganusov et al., 2005; Boer et al., 2006). Furthermore, these latter models often include parameters for transitioning to the memory phase through a second, slower rate of loss, and this has not been implemented into the Cyton framework yet as the mode of transition is not known. The difference between fitting parsimonious models to *in vitro* and *in vivo* data may eventually be reconciled as the community continues to improve methods that introduce differentiation, memory formation and reveal additional features of responding cell phenotype, such as cell cycle status, as originally envisaged by Antia et al. (2003).

## 5 Methods

### 5.1 Mice

All mice were maintained under specific pathogen-free conditions in the WEHI animal facilities (Parkville, Victoria, Australia) and used at 5–12 weeks of age. All experiments were performed under the approval of the WEHI Animal Ethics Committee. FUCCI red/green (RG) mice were acquired by crossing FUCCI Red (B6.B6D2-Tg (FUCCI)639Bsi) with FUCCI Green (B6.B6D2-Tg (FUCCI)492Bsi) mice, both obtained from Riken BioResource Centre (Sakaue-Sawano et al., 2008). FUCCI RG mice were then crossed to OT-I or OT-I/Ly5.1 mice to obtain OT-I/FUCCI RG and OT-I/FUCCI RG-Ly5.1 respectively (Dowling et al., 2014). In one experiment (stimulation with N4, *α*CD28 and IL-12) cells from C57BL/6 mice that were irradiated and reconstituted with bone marrow from OT-I/FUCCI RG-Ly5.1 were used.

### 5.2 CD8^+^ T Cell Isolation

OT-I CD8^+^ T cells were isolated from single cell suspensions prepared from lymph nodes (axillary, branchial, inguinal) by negative selection using EasySep Mouse CD8*α* ^+^ T cell Isolation kit (StemCell technologies) according to the manufacturer’s protocol. Purity (CD8^+^ V*α*2^+^) was typically between 80 and 95%. Splenocytes were used for the isolation of CD8^+^ V*α*2^+^Ly5.1^+^ T cells from C57BL/6 mice that were irradiated and reconstituted with bone marrow from OT-I/FUCCI RG-Ly5.1. Purified CD8^+^ T cells were labelled with 5 *μ*M CellTrace™ Violet (CTV, Invitrogen) to track and monitor cell division in parallel bulk cultured by flow cytometry. Labelling was performed for 20 min at 37°C in PBS + 0.1% BSA.

### 5.3 *In Vitro* Cell Culture

All T cell cultures were prepared using filming medium (GIBCO advanced RPMI 1640 without phenol red + 5% GIBCO FCS) at 37°C and 5% CO_2_ in a humidified atmosphere. All cell cultures contained 25 *μ*g/ml anti-mouse IL-2 antibody (S4B6: WEHI antibody facility) which neutralises mouse IL-2 but does not recognise human IL-2 (Deenick et al., 2003).

Cells were either stimulated with plate bound anti-CD3 (*α*CD3: WEHI antibody facility, clone 145-2C11: 10 *μ*g/ml) or with the peptide for the OT-I TCR, SIINFEKL (N4) (Auspep) at 0.01 *μ*g/ml.

For stimulation with *α*CD3, CD8^+^ T cells were cultured on 24-well plates coated with *α*CD3 at 40,000 cells in 1 ml per well in the presence of 1, 3.16, or 10 U/ml recombinant human IL-2 (Peprotech) and 25 *μ*g/ml S4B6. After 24 h of culture cells were harvested, washed twice with filming medium, counted and resuspended at 5,000–10,000 cell/ml in filming medium supplemented with 25 *μ*g/ml S4B6 and 1, 3.16, or 10 U/ml recombinant human IL-2. The units of IL-2 were abbreviated to 1U, 3U and 10U for each concentration throughout the paper.

In experiments using N4 peptide CD8^+^ T cells were cultured with 0.01 *μ*g/ml N4 at 2 × 10^4^ cells per ml in 200 *μ*L of a 96-well U-bottom plate in the presence of 25 *μ*g/ml S4B6. 2 *μ*g/ml *α*CD28 (clone 37.51, WEHI antibody facility) or 1 ng/ml IL-12 (Peprotec) were added as indicated. Cells were cultured for 24 h, washed and resuspended at 5,000–10,000 cells/ml in filming medium containing 25 *μ*g/ml S4B6.

For one experiment CD8^+^ T cells cultured for 24 h with N4 in presence or absence of *α*CD28 were split and supplemented or not with 1 U/ml rhIL-2 before replating for filming.

In one experiment CD8^+^ T cells were cultured with N4 alone and addition of either *α*CD28, IL-12, or both for 24 h, then washed, resuspended, and replated for filming without any further stimuli added.

For filming, 250 *μ*L cell suspension was added per chamber of an 8 well *μ*-Slide chamber (Ibidi) containing 125 *μ*m (MGA-125-01) or 70 *μ*m (MGA-7-01) microgrids (Daniel Day, Microsurfaces). These conditions resulted in a significant portion of microwells containing 1 cell per well. Before the start of filming, cells were incubated for ≈ 2 h at 37°C with 5% CO_2_ in a humidified atmosphere. Slide chambers were then transferred to an environmentally controlled microscope (Carl Zeiss) and incubated at 37°C with 5% CO_2_ in a humidified atmosphere.

### 5.4 Live Cell Imaging and Cell Tracking

For single cell filming, microgrids (70/125*μ*m, Daniel Day, Microsurfaces) were placed into an 8 well chamberslide (Ibidi *μ*-slide). Chambers were UV sterilised with 40 *μ*L 100% ethanol in a laminar-flow cabinet for at least 30 min until dry. Another 40 *μ*L ethanol was added and rinsed 10x with filming medium (advanced RPMI 1640 without phenol red). 250*μ*L filming medium was added to each chamber and left in the incubator at 37°C overnight to dissolve air bubbles. To reduce background fluorescence of the medium, chambers (grids) were bleached for 2 h using a 470 nm LED, just prior to adding the cells.

Live cell imaging was conducted on an environmentally controlled 37°C + 5% CO2 humidified Zeiss Axiovert 200 M microscope. Brightfield images were captured with a Zeiss AxioCam MRm (1.4 megapixels) atttached to a 0.63x C-mount, using a Plan-Apochromat 20x objective (0.8 n.a.). A GFP/DsRed-A (Semrock) filter block (excitation LED 470/555 nm set at 25%/100%, respectively, with an exposure time of 200*μ*s) was used for detecting green and red fluorescence. Red, green, bright-field and out-of-focus images were taken at 165 s intervals for 5 or 6 days. Bright-field and fluorescent raw images of single cells in microgrids were digitally processed resulting in overlaid red/green images corrected for background noise of the medium.

Cell tracking was performed using the image processing package FIJI (Schindelin et al., 2012). Lineage Tracker plug-in was used for cell segmentation and tracking (Downey et al., 2011). Gaps or mistakes in segmentation and tracking were adjusted manually to ensure data accuracy. Cells in each well were followed until they either died, indistinguishable from nearby cells or the experiment ended.

For CD8^+^ T cell data in the presence of IL-2, measurements from two independent experiments were aggregated.

### 5.5 Data Selection and Tree Collapse

For each family tree $c\in N_{\geq 0}$ , the times to divide ${\left\{T_{div}^{x}\right\}}_{c}$ , to die ${\left\{{T_{die}}^{x}\right\}}_{c}$ and to loss ${\left\{T_{loss}^{x}\right\}}_{c}$ of all cells were recorded using time-lapse microscope. All of these variables were measured from *t* = 0*h*, i.e., the beginning of the experiment. *T*_*loss*_ is defined as the time at which the cell becomes indistinguishable to the nearby cells, or survives until the end of given experiment time frame, thus, were lost from the experiment. In order to keep track of the cells’ relation, a unique label was given to each cell by *x*. Let $X_{c}$ be the collection of all *x* for a family *c*, where $x=\left\langle x_{1},x_{2},\ldots ,x_{j}\right\rangle$ with *x*_*j*_ ∈ {1, 2} is a finite and ordered sequence of 1 and 2 s. Beginning with a founder cell, defined as $x=\left\langle 0\right\rangle$ , we denote its first and second daughter cells in generation 1 by $x=\left\langle 1\right\rangle$ and $x=\left\langle 2\right\rangle$ , respectively. In general, $\left\langle x_{1},x_{2},\ldots ,x_{j}\right\rangle$ represents the $x_{j}^{\text{th}}$ daughter of the … of the $x_{2}^{\text{th}}$ daughter of the $x_{1}^{\text{th}}$ daughter of the founder cell (see Harris, 1963, Ch.6). For example, $x=\left\langle 1,1,2\right\rangle$ denotes the second daughter of the first daughter of the first daughter of the founder cell. Given a unique identifier of the cell, the generation *k* is noted *g*(*x*): = *k* with $g\left(\left\langle 0\right\rangle \right)=0$ . With this construct, we define the raw measurement of times as a set $T_{c}=\left\{T_{div}^{x},T_{die}^{x},T_{loss}^{x}:x\in X_{c}\right\}$ .

For the analyses in Section 2.2 and Section 2.4, we filtered for families that had at least divided once and satisfied the condition $\max\left(T_{c}\right)=T_{die}^{x}$ . In essence, we eliminated incomplete family trees that contain unusually long-surviving cells, but allowed lost cells to be in place as long as the last observed event is death in a given family. Indeed, there is an increasing chance of observing more lost cells as the family gets larger. However, it was previously shown that the regularity of a family is a result of correlated cell divisions as a biological feature inherited within the family even when considering the unrecovered samples (Marchingo et al., 2016). Therefore, it is highly likely that the lost cells due to indistinguishable circumstances might had undergone similar fates with its sibling, thereby maximising the number of data points while reducing any potential selection bias, whereas it is difficult to weigh how including the long-surviving cells might affect all the other analyses. We noted the long-surviving and “no division” cells constitute approximately 23 and 29% on average, respectively, across all the experiments (data not shown).

Given the heritable feature, we summarise a family tree by collapsing it to a single representative line (Figure 1C). By collapsing, we mean substitute average time to divide (and to die) of the cells in a given generation *k*. We also enumerated all dead cells within a family and calculated mean time to last division (*T*_*ld*_) as a proxy to the division destiny time. In summary, we represent a single family by $T\left(c\right)=\left(T_{div}^{0},\ldots ,T_{div}^{k},T_{die}^{0},\ldots ,T_{die}^{k}\right)$ so long as we observed division or death events in each generation *k*. Table 3 shows the number of retained clones used in all analyses presented in this paper after applying the filtering rule.

**TABLE 3:** Number of clones used in the analysis of the filming datasets. The numbers were obtained by filtering on clones that had divided at least once and whose last event was a death event (not a loss or a division). The percentage is expressed in relation to the total number of clones in the experiment. For a given cell type and stimulation, the values of each Cyton2 variable were extracted for the statistical analyses.

Cell type	Stimulation	Number of clones			
		Time to first div. ( $T_{div}^{0}$ )	Subsequent div. Time ( $T_{div}^{k}$ )	Time to last div. (*T*_*ld*_)	Time to death (*T*_*die*_)
B (B-exp1)	CpG	69 (63.9%)	56 (51.9%)	69 (63.9%)	69 (63.9%)
B (B-exp2)	CpG	73 (83.0%)	63 (71.6%)	73 (83.0%)	73 (83.0%)
CD8^+^ T	1U IL-2	28 (25.7%)	4 (3.7%)	28 (25.7%)	28 (25.7%)
	3U IL-2	34 (37.8%)	13 (14.4%)	34 (37.8%)	34 (37.8%)
	10U IL-2	50 (30.7%)	16 (9.8%)	50 (30.7%)	60 (30.7%)
CD8^+^ T (T-exp1)	N4	20 (44.4%)	0 (0%)	20 (44.4%)	20 (44.4%)
	N4 + *α*CD28	19 (46.3%)	1 (2.4%)	19 (46.3%)	19 (46.3%)
	N4 + IL-2	28 (75.7%)	4 (10.8%)	28 (75.7%)	28 (75.7%)
	N4 + *α*CD28 + IL-2	33 (89.2%)	13 (35.1%)	33 (89.2%)	33 (89.2%)
CD8^+^ T (T-exp2)	N4	27 (73.0%)	12 (32.4%)	27 (73.0%)	27 (73.0%)
	N4 + *α*CD28	17 (44.7%)	12 (31.6%)	17 (44.7%)	17 (44.7%)
	N4 + IL-12	13 (44.8%)	9 (31.0%)	13 (44.8%)	13 (44.8%)
	N4 + *α*CD28 + IL-12	11 (30.6%)	7 (19.4%)	11 (30.6%)	11 (30.6%)

### 5.6 Agent-Based Model

We developed an agent-based model (ABM) to simulate cells in a single family with the correlated structure proposed for the Cyton2 model. Each realisation of the simulation represents one clonal family. Upon initialisation, the founder cell is assigned time to first division, global destiny and global death times, which are drawn randomly from three independent lognormal distributions. Also, the subsequent division times are randomly sampled for each generation from a lognormal distribution, but the progeny cells of the same generation share the division time. If the founder cell reaches time to first division, it creates two daughter cells, which inherit global destiny and death times. If the cell reaches its division destiny, we immediately classify it as a destiny cell and prevent it from further division. When the cells reach death time, they are removed from the simulation. The model was implemented in Python (version 3.8.6).

### 5.7 Statistical Analysis: Bayesian Framework

In Section 2.2, 2.3, the correlations of all possible pairs between time to first division ( $T_{div}^{0}$ ), average subsequent division time ( $T_{div}^{k\geq 1}$ ), time to last division (*T*_*ld*_) and time to death (*T*_*die*_) were estimated using Bayesian inference. For a given pair of variables and its observed data, say $d_{i}\in D=\left\{\left(x_{i},y_{i}\right):i=1,2,\ldots ,n\right\}$ where *n* is the number of observations, we used bivariate normal distribution to estimate the correlation coefficient (*ρ*). This entails $x_{i}∼N\left(\mu_{x},\sigma_{x}\right)$ and $y_{i}∼N\left(\mu_{y},\sigma_{y}\right)$ . With uninformative priors on the hyper-parameters *μ*_*x*_, *μ*_*y*_ ∼ *U* (0, 1000), *σ*_*x*_, *σ*_*y*_ ∼ *U* (0, 1000) and *ρ* ∼ *U* (−1, 1), we define the bivariate normal distribution.

$d_{i}∼N\left(\mu ,\Sigma \right),$

where ***μ*** = (*μ*_*x*_, *μ*_*y*_) is a vector of means for *x*_*i*_ and *y*_*i*_, and $\Sigma =\left[\begin{array}{cc} \sigma_{x}^{2} & \rho \sigma_{x}\sigma_{y}\\ \rho \sigma_{x}\sigma_{y} & \sigma_{y}^{2} \end{array}\right]$ is a covariance matrix. We used an extension of the Hamiltonian MCMC algorithm, No-U-Turn Sampler (Hoffman and Gelman, 2014), implemented in PyMC3 (version 3.9.3) (Salvatier et al., 2016) to obtain the marginal posterior distributions of *ρ*, *μ*_*x*_, *σ*_*x*_, *μ*_*y*_, *σ*_*y*_. Given these distributions, we calculated 95% credible interval for *ρ*, and 90, 95, and 99% density regions of (*x*, *y*). In addition, we formulated Bayesian hypothesis testing, where the null hypothesis is *H*_0_: *ρ* = 0 and alternative hypothesis is *H*_1_: *ρ* ≠ 0 (which translates to *H*_1_: *ρ* ∼ *U* (−1, 1)) (Jeffreys, 1961). This is formally stated as a ratio of likelihoods of hypotheses given the data,

$$\frac{P\left(H_{0}|D\right)}{P\left(H_{1}|D\right)}=\frac{P\left(H_{0}\right)}{P\left(H_{1}\right)}\times \frac{P\left(D|H_{0}\right)}{P\left(D|H_{1}\right)}.$$

In order to grade if the data is more probable under *H*_0_ or *H*_1_, the Bayes factor ${BF}_{01}=P\left(D|H_{0}\right)/P\left(D|H_{1}\right)$ was used given priors of *P*(*H*_0_) and *P*(*H*_1_). When *H*_1_: *ρ* ∼ *U* (−1, 1), it can be computed by evaluating the following integral (Jeffreys, 1961; Wagenmakers et al., 2016):

$${BF}_{01}=1/{BF}_{10},\text{ where }{BF}_{10}=\frac{1}{2}\int_{-1}^{1}\frac{{\left(1-\rho^{2}\right)}^{\frac{n-1}{2}}}{{\left(1-\rho r\right)}^{n-\frac{3}{2}}}d\rho ,$$

where *r* denotes for the sample correlation defined as $r=\sum_{i=1}^{n}\left(x_{i}-\bar{x}\right)\left(y_{i}-\bar{y}\right)/\sqrt{\sum_{i=1}^{n}{\left(x_{i}-\bar{x}\right)}^{2}\sum_{i=1}^{n}{\left(y_{i}-\bar{y}\right)}^{2}}$ . For the interpretation of Bayes factor, we adopted the discrete categories of evidential strength proposed in Jeffreys (1961) (Table 4).

**TABLE 4:** Bayes factor interpretation.

Bayes factor: BF_01_ (BF_10_)	Interpretation
>100	Extreme evidence for *H*_0_ (*H*_1_)
30–100	Very strong evidence for *H*_0_ (*H*_1_)
10–30	Strong evidence for *H*_0_ (*H*_1_)
1–3	Anecdotal evidence for *H*_0_ (*H*_1_)
1	No evidence

In Section 2.4, six probability distributions were assessed for $T_{div}^{0},T_{div}^{k\geq 1},T_{ld},T_{die}$ under the Bayesian framework in a similar manner to estimating the correlation coefficient. Table 5 shows the list of candidate distributions and the uninformative priors prescribed for respective hyper-parameters.

**TABLE 5:** List of candidate parametric distribution classes.

Candidates	Priors	Target distribution
A	*α*_*G*_, *β*_*G*_ ∼ *U* (0, 200)	$T_{div}^{0},T_{div}^{k\geq 1},T_{ld},T_{die}∼Gamma\left(\alpha_{G},\beta_{G}\right)$
B	*m*, *s* ∼ *U* (0, 200)	$T_{div}^{0},T_{div}^{k\geq 1},T_{ld},T_{die}∼LN\left(m,s\right)$
C	*μ*, *σ* ∼ *U* (0, 200)	$T_{div}^{0},T_{div}^{k\geq 1},T_{ld},T_{die}∼N\left(\mu ,\sigma \right)$
D	*α*_*W*_, *β*_*W*_ ∼HalfNormal (500)	$T_{div}^{0},T_{div}^{k\geq 1},T_{ld},T_{die}∼Weibull\left(\alpha_{W},\beta_{W}\right)$
E	*λ* ∼ *U* (0, 2), *c* ∼ *U* (0, *∞*)	$T_{div}^{0},T_{div}^{k\geq 1},T_{ld},T_{die}∼Delayed\;Exp\left(\lambda ,c\right)$
F	*m*_*d*_, *s*_*d*_ ∼ *U* (0, 200), *c* ∼ *U* (0, *∞*)	$T_{div}^{0},T_{div}^{k\geq 1},T_{ld},T_{die}∼Delayed\;LN\left(m_{d},s_{d},c\right)$

Given posterior distributions of the parameters, we adopted WAIC (Watanabe and Opper, 2010) score to quantitatively assess the candidates, which is estimated as follows:

$$WAIC\left(z,\Theta \right)=-2\left(\overset{n}{\underset{i=1}{\sum }}\log\left[\frac{1}{S}\overset{S}{\underset{s=1}{\sum }}P\left(z_{i}|\Theta_{s}\right)\right]-\overset{n}{\underset{i=1}{\sum }}{\mathrm{Var}}_{s=1}^{S}\log\left(P\left(z_{i}|\Theta_{s}\right)\right)\right),$$ (2)

where *z* is the data with *n* independent number of observations, Θ is the posterior distribution, Θ_*s*_ is the *s*th set of sampled parameter values in the posterior distribution with *S* number of samples and ${\mathrm{Var}}_{s=1}^{S}a_{s}=\frac{1}{S-1}\sum_{s=1}^{S}{\left(a_{s}-\bar{a}\right)}^{2}$ denotes for the sample variance (see McElreath, 2020, Ch.7; Vehtari et al., 2017). The first and the second terms in Eq. 2 are known as the log-pointwise-predictive-density (lppd) and the penalty term, respectively. For direct comparison of the candidates, we computed the standard error by calculating the variance over the individual observations instead of their summation under the assumption of normality of WAIC.

$$se\left(WAIC\right)=\sqrt{n\times {\mathrm{Var}}_{i=1}^{n}\left(-2\left(\log\left[\frac{1}{S}\overset{S}{\underset{s=1}{\sum }}P\left(z_{i}|\Theta_{s}\right)\right]-{\mathrm{Var}}_{s=1}^{S}\log\left(P\left(z_{i}|\Theta_{s}\right)\right)\right)\right)}.$$

Let us denote WAIC_*i*_ to be the term in $\sqrt{\left(\cdot \right)}$ such that $WAIC=\sum_{i=1}^{n}{WAIC}_{i}$ , then the standard error of the difference of WAIC between, for instance, candidate A and B can be calculated,

$$se\left({WAIC}^{A}-{WAIC}^{B}\right)=\sqrt{n\times {\mathrm{Var}}_{i=1}^{n}\left({WAIC}_{i}^{A}-{WAIC}_{i}^{B}\right)}.$$

### 5.8 Equations for Dynamic Evolution of the Mean

In commonly employed division diluting dye experiments, individual families are not observed and initial cell numbers are typically in their thousands suggesting the use of mean system behaviour as an appropriate descriptor. Thus to fit the model to such data we derive equations for the mean population dynamics per generation for Cyton2.

Let *Z*_*g*_(*t*) denote the number of cells alive in generation *g* ∈ {0, 1, *…* , *G*} at time *t* ≥ 0. Then, *Z*_*g*_(*t*) can be expressed with the variables shown in Section 2.1 for any chosen probability density functions for the random variables. Here, we separately derived $E\left[Z_{g}\left(t\right)\right]$ for *g* = 0 and *g* > 0 cases as lymphocytes generally take longer to divide for the first time than at later generations. In essence, we begin the derivation with parameters $\theta =\left(T_{div}^{0},{\left\{M_{g}\right\}}_{g\geq 1},T_{dd},T_{die}\right)$ denoting time to first division, subsequent division time per generation, time to destiny and time to death, respectively.

#### Generation Zero

We assume we are following the activation dynamics of a set of resting cells and these cells are provided with signals that program a limited proliferative response. For the purposes here, we also assume that all cells are activated at time *t* = 0, erasing the prior cell programming and survival characteristics. Situations where only a proportion of cells are activated, or where the activated cells take some extended time to transition to the new programming, leading to some early cell death are useful modifications suited to particular applications. Such modifications are discussed further in Supplementary Material.

For a given family tree, the number of live cells dividing, dying or reaching destiny in generation *g* = 0 at time *t* is given by

$$Z_{0}\left(t\right)=1_{\left\{T_{die}>t\right\}}1_{\left\{\min\left(t,T_{dd}\right)<T_{div}^{0}\right\}},$$ (3)

where 1 is an indicator function. Assuming that the random variables *T*_*die*_, *T*_*dd*_ and $T_{div}^{0}$ are independent of each other as we established in Section 2.2, the expected number is given by

$$E\left[Z_{0}\left(t\right)\right]=P\left(T_{die}>t\right)P\left(\left\{\min\left(t,T_{dd}\right)<T_{div}^{0}\right\}\right).$$

We expand the second term, and by the law of total probability we obtain

$$P\left(\left\{\min\left(t,T_{dd}\right)<T_{div}^{0}\right\}\right)=P\left(T_{div}^{0}>t\right)P\left(T_{dd}>t\right)+P\left(T_{div}^{0}>T_{dd}\right)P\left(T_{dd}\leq t\right).$$

Thus, the expected number of cells in generation zero is,

$$E\left[Z_{0}\left(t\right)\right]=P\left(T_{die}>t\right)\left[P\left(T_{div}^{0}>t\right)P\left(T_{dd}>t\right)+\int_{0}^{t}dP\left(T_{dd}\leq \tau \right)P\left(T_{div}^{0}>\tau \right)\right].$$ (4)

This equation can be interpreted as follows: a cell in generation zero remains alive when *T*_*die*_ > *t*, and it is sorted either in initial state or in destiny state. The cell in the initial state can divide, reach destiny or die whichever event comes first. However, the destiny cell can no longer divide but only awaits for death.

#### Generations **>** 0

To calculate the expected number of live cells for *g* > 0, we limit the windows of cells being in generation *g* by constraining with $t\in \left[T_{div}^{0}+\sum_{k=1}^{g-1}M_{k},T_{div}^{0}+\sum_{k=1}^{g}M_{k}\right)$ , that is

$$Z_{g}\left(t\right)=2^{g}1_{\left\{T_{die}>t\right\}}1_{\left\{T_{div}^{0}+\overset{g-1}{\underset{k=1}{\sum }}M_{k}\leq \min\left(t,T_{dd}\right)<T_{div}^{0}+\overset{g}{\underset{k=1}{\sum }}M_{k}\right\}}.$$ (5)

The factor 2^*g*^ is required to include the effect of clonal expansion of the cells that have divided *g* times. Assuming *T*_*die*_, *T*_*dd*_, *M*_*k*_ and $T_{div}^{0}$ are independent of each other, the expected value is

$$E\left[Z_{g}\left(t\right)\right]=2^{g}P\left(T_{die}>t\right)P\left(T_{div}^{0}+\overset{g-1}{\underset{k=1}{\sum }}M_{k}\leq \min\left(t,T_{dd}\right)<T_{div}^{0}+\overset{g}{\underset{k=1}{\sum }}M_{k}\right).$$

Defining $X_{g}=T_{div}^{0}+\sum_{k=1}^{g-1}M_{k}$ and *Y*_*g*_ = *M*_*g*_, then, similarly to the *g* = 0 case, we expand and employ the law of total probability to obtain

$$\begin{array}{ll} P\left(X_{g}\leq \min\left(t,T_{dd}\right)<X_{g}+Y_{g}\right)= & P\left(X_{g}\leq t<X_{g}+Y_{g}\right)P\left(T_{dd}>t\right)\\ & +P\left(X_{g}<T_{dd}<X_{g}+Y_{g}\right)P\left(T_{dd}\leq t\right). \end{array}$$

Hence, the expected number of cells in *g* > 0 is

$$\begin{array}{cc} E\left[Z_{g}\left(t\right)\right] & =2^{g}P\left(T_{die}>t\right)\times \\ & \left[P\left(T_{dd}>t\right)\int_{0}^{t}dP\left(X_{g}\leq \tau \right)P\left(Y_{g}>t-\tau \right)+\int_{0}^{t}dP\left(T_{dd}\leq \tau \right)P\left(X_{g}<\tau <X_{g}+Y_{g}\right)\right]. \end{array}$$ (6)

Together with Eqs 4, 6, we can calculate the average number of live cells for a family in generation *g* at time *t* for any distribution class of $T_{div}^{0},{\left\{M_{g}\right\}}_{g\geq 1},T_{dd}$ and *T*_*die*_. Since the equations are equally applicable for *N*_0_ number of initial founder cells, we generalise these by multiplying *N*_0_ such that

$$y_{g}\left(t;\theta \right):=E\left[N_{0}Z_{g}\left(t;\theta \right)\right]=N_{0}E\left[Z_{g}\left(t;\theta \right)\right],$$

where $\theta =\left(T_{div}^{0},{\left\{M_{g}\right\}}_{g\geq 1},T_{dd},T_{die}\right)$ are the parameters of the Cyton2 model. Typically, the random variables are equipped with a lognormal distribution, which has two additional parameters, thus, we have total of 6 + 2*g* free parameters to estimate.

#### Reduced Cyton2 Model

To fit FACS data, we simplify the model by assuming that the subsequent division time is a constant rather than a set of random variables, that is, $M_{g}=m\in R_{>0}$ for all *g* > 0. This is based on the empirical observation made from filming data that, after the first division, the cells divide at a consistent rate with little inter- and intra-clonal variability (Figures 2A2,B2). This step drastically reduces the number of free parameters, and it no longer depends on the number of generations but purely on the choices of probability density function of $T_{div}^{0},T_{dd}$ and *T*_*die*_. Essentially, the reduced model has $\tilde{\theta }=\left(T_{div}^{0},m,T_{dd},T_{die}\right)$ parameters. Since Eq. 4 does not depend on the subsequent division time, it remains the same:

$$E\left[{\tilde{Z}}_{0}\left(t\right)\right]=E\left[Z_{0}\left(t\right)\right]=P\left(T_{die}>t\right)\left[P\left(T_{div}^{0}>t\right)P\left(T_{dd}>t\right)+\int_{0}^{t}f_{T_{dd}}\left(\tau \right)P\left(T_{div}^{0}>\tau \right)d\tau \right],$$

where $f_{T_{dd}}$ is the probability density function of *T*_*dd*_. However, Eq. 6 can be further simplified to

$$\begin{array}{cc} & E\left[{\tilde{Z}}_{g}\left(t\right)\right]=2^{g}P\left(T_{die}>t\right)\times \\ & \left[P\left(T_{dd}>t\right)P\left(t-gm<T_{div}^{0}<t-\left(g-1\right)m\right)+\int_{0}^{t}f_{T_{dd}}\left(\tau \right)P\left(\tau -gm<T_{div}^{0}<\tau -\left(g-1\right)m\right)d\tau \right]. \end{array}$$

We used the reduced Cyton2 model for all our analyses of FACS data presented in this paper.

$${\tilde{y}}_{g}\left(t;\tilde{\theta }\right):=N_{0}E\left[{\tilde{Z}}_{g}\left(t;\tilde{\theta }\right)\right].$$ (7)

### 5.9 Fitting the Cyton2 Model

Division structured population datasets obtained from FACS were fitted to the reduced Cyton2 model (i.e. Eq. 7). In total, there are 7 parameters to be estimated for each dataset assuming that the random variables are lognormally or normally distributed, thus if we have *N* number of conditions, we have a maximum of 7*N* free parameters to be fitted. For all conditions, we always used cell numbers at the beginning of the stimulus (typically at *t* = 0) as a fixed initial cell number.

For each set of cell numbers {*n*_*g*_,_*r*_ (*t*_*i*_)} from the data, where *i* ∈ {0, 1, *…* , *I*}, *g* ∈ {0, 1, *…* , *G*} and *r* ∈ {0, 1, *…* , *R*} are time, generation and replicate indices, respectively, we obtained point estimates of the parameters. To achieve this, we used least-squares method with Levenberg-Marquardt (Marquardt, 1963) optimisation algorithm implemented in Python library LMFIT (version 1.0.2) (Newville et al., 2014). We defined the residual sum of squares (RSS) as our cost function,

$$C\left(\tilde{\theta }\right)=\overset{I}{\underset{i=0}{\sum }}\overset{G}{\underset{g=0}{\sum }}\overset{R}{\underset{r=0}{\sum }}{\left(n_{g,r}\left(t_{i}\right)-{\tilde{y}}_{g}\left(t_{i};\tilde{\theta }\right)\right)}^{2},$$

such that we find an approximate minimum,

$$\left\{{\tilde{\theta }}^{*}\right\}\in \underset{\tilde{\theta }}{\mathrm{arg min}}C\left(\tilde{\theta }\right).$$

As the algorithm requires a set of starting parameter values, we prescribed 100 sets of initial values drawn uniformly at random from the appropriate parameter ranges, and recorded RSS for each set to identify the best fitted parameters by the lowest RSS. For fitting multiple datasets simultaneously, which requires an extra sum over all datasets in the cost function, the algorithm needs to explore higher dimension of the parameter space compared to fitting one dataset at each iteration. Therefore, we used 200 sets of initial values to increase range of the exploration. After identifying the best fit, we performed bootstrap method (Efron, 1979) with an artificial dataset that was resampled with replacement (per time point) from the original measured data. We repeated this process 1,000 times, which resulted in 1,000 additional estimates for each parameter. This allowed us to calculate 95% confidence intervals on the best fitted parameter values. Additionally, we also obtained confidence bands for extrapolated cell numbers by calculating 95% percentile range at each of discretised time point from the model.

## Data Availability

The datasets presented in this study can be found in online repositories. The names of the repository/repositories and accession number(s) can be found below: https://github.com/hodgkinlab/cyton2-paper.
