# Supplementary material for: Cyton2: A Model of Immune Cell Population Dynamics That Includes Familial Instructional Inheritance
Source: Front Bioinform. 2021 Oct 26;1:723337. doi: 10.3389/fbinf.2021.723337 (PMC9581048; doi:10.3389/fbinf.2021.723337)
Supplement: Supplementary file 1 [file DataSheet1.PDF]

## Supplementary Information

**Modifications for calculating number of cells in generation zero**. In Methods Section 5.8 we derive a formula for calculating expected number of cells in generation zero, assuming cells are immediately programmed upon activation to adopt random variables (RVs) for Cyton2 evolution. Here we offer a series of revised formulas for immune activation situations where the calculation of cells in generation zero is more complex.

**Partial activation, no death**. In many culture arrangements, only a fraction of the cells receive the activation stimulus and adopt the new program governed by Cyton2 parameters. Here the population is first divided into fractions. The number of stimulated cells is given as $N_0 \cdot p$, where $p \in [0, 1]$ is stimulated cell fraction, and is treated as in Methods Section 5.8 to calculate overall expected number of cells. The unstimulated cells, $N_0(1-p)$, remain undivided. This introduces one new parameter, $p$.

In essence, it is equivalent of introducing a Bernoulli RV, $A : \Omega \mapsto \{0, 1\}$, that represents an event of activation for the founder cell in a family tree, where $\mathbb{E}[A] = p \in [0, 1]$ is the probability of activation. Then, the number of cells in a given family $Z_g(t)$ (c.f. Eq. (3) and (5)) needs to be modified to

$$Z_g(t) = \begin{cases} (1-A) + A\mathbb{1}_{\{T_{die}>t\}}\mathbb{1}_{\{\min(t,T_{dd})<T^0_{div}\}} & \text{for } g = 0 \\ 2^g A\mathbb{1}_{\{T_{die}>t\}}\mathbb{1}_{\{T^0_{div}+\sum_{k=1}^{g-1} M_k \le \min(t,T_{dd}) < T^0_{div}+\sum_{k=1}^{g} M_k\}} & \text{for } g > 0 \end{cases} \quad \text{(S1)}$$

**Partial activation, death program**. Here the arrangement is as above, but the unstimulated cells will die according to a distinct probability. To do so, we need an extra RV for the unstimulated time to death distribution, $T_U$. After splitting the population into the fractions by parameter $p$, the unstimulated cells remain in generation zero, but die according to distribution of $T_U$. Similar to Eq. (S1), the modified equation will be

$$Z_g(t) = \begin{cases} (1-A)\mathbb{1}_{\{T_U>t\}} + A\mathbb{1}_{\{T_{die}>t\}}\mathbb{1}_{\{\min(t,T_{dd})<T^0_{div}\}} & \text{for } g = 0 \\ 2^g A\mathbb{1}_{\{T_{die}>t\}}\mathbb{1}_{\{T^0_{div}+\sum_{k=1}^{g-1} M_k \le \min(t,T_{dd}) < T^0_{div}+\sum_{k=1}^{g} M_k\}} & \text{for } g > 0 \end{cases} \quad \text{(S2)}$$

**Slow reprogramming**. In a common situation, cells in culture take some time to integrate the activation signals and reprogram survival from the unstimulated fate. The signals leading to reprogramming are in a race with the initial survival program of the cells. For this we define an activation time for completion of the program and assume that cells behave as if unstimulated to that activation time. If adopting this method, the activation time becomes time zero for the calculation of PDFs/CDFs of RVs. This scenario can also occur when only a fraction of cells are stimulated in culture. Hence, calculation of cells requires values for $p$, $t_A \in \mathbb{R}_{>0}$ (activation time), and $T_U$ according to

$$Z_g(t) = \begin{cases} \mathbb{1}_{\{T_U>t\}} & \text{for } t \le t_A, g = 0 \\ \mathbb{1}_{\{T_U>t_A\}}\left[(1-A)\mathbb{1}_{\{T_U>t\}} + A\mathbb{1}_{\{X_{die}>t\}}\mathbb{1}_{\{\min(t,X_{dd})<X^0_{div}\}}\right] & \text{for } t > t_A, g = 0 \\ \mathbb{1}_{\{T_U>t_A\}}\left[2^g A\mathbb{1}_{\{X_{die}>t\}}\mathbb{1}_{\{X^0_{div}+\sum_{k=1}^{g-1} M_k \le \min(t,X_{dd}) < X^0_{div}+\sum_{k=1}^{g} M_k\}}\right] & \text{for } t > t_A, g > 0 \end{cases} \quad \text{(S3)}$$

where $X^0_{div}, X_{dd}, X_{die} : \Omega \mapsto (t_A, \infty)$ are shifted RVs such that $X = T + t_A$. Note that Eq. (S3) reduces to Eq. (S2) when $t_A = 0$.

# Supplementary Figures and Tables

| Cell Type | Stimulation | Initial # clones | Observed cells: # divided cells (# dead cells) [# lost cells] | | | | | | | |
|---|---|---|---|---|---|---|---|---|---|---|
| | | | Gen 0 | Gen 1 | Gen 2 | Gen 3 | Gen 4 | Gen 5 | Gen 6 | Gen 7 |
| B | CpG (B-exp1) | 108 | 108 (0) [0] | 169 (30) [17] | 204 (67) [67] | 153 (147) [108] | 82 (134) [90] | 37 (95) [32] | 1 (58) [15] | 0 (0) [2] |
| | CpG (B-exp2) | 88 | 88 (0) [0] | 145 (24) [7] | 150 (99) [41] | 175 (101) [24] | 75 (203) [72] | 18 (93) [39] | 2 (18) [16] | 0 (4) [0] |
| $CD8^+$ T | 1U IL-2 | 109 | 29 (79) [1] | 6 (50) [2] | 0 (12) [0] | - | - | - | - | - |
| | 3U IL-2 | 90 | 68 (22) [0] | 77 (48) [11] | 46 (35) [73] | 2 (4) [86] | 4 (0) [0] | 1 (0) [7] | 0 (0) [2] | - |
| | 10U IL-2 | 163 | 101 (62) [0] | 122 (71) [9] | 94 (59) [91] | 12 (12) [164] | 0 (0) [24] | - | - | - |
| $CD8^+$ T (T-exp1) | N4 | 45 | 20 (25) [0] | 0 (40) [0] | - | - | - | - | - | - |
| | N4 + $\alpha$CD28 | 41 | 22 (19) [0] | 8 (36) [0] | 0 (4) [12] | - | - | - | - | - |
| | N4 + IL-2 | 37 | 28 (9) [0] | 6 (50) [0] | 0 (12) [0] | - | - | - | - | - |
| | N4 + $\alpha$CD28 + IL-2 | 47 | 36 (11) [0] | 31 (41) [0] | 20 (38) [4] | 0 (19) [21] | - | - | - | - |
| $CD8^+$ T (T-exp2) | N4 | 37 | 30 (7) [0] | 27 (33) [0] | 12 (34) [8] | 0 (16) [8] | - | - | - | - |
| | N4 + $\alpha$CD28 | 38 | 33 (5) [0] | 52 (14) [0] | 8 (30) [66] | 0 (16) [0] | - | - | - | - |
| | N4 + IL-12 | 29 | 22 (7) [0] | 33 (11) [0] | 12 (18) [36] | 5 (17) [2] | 0 (6) [4] | - | - | - |
| | N4 + $\alpha$CD28 + IL-12 | 36 | 32 (4) [0] | 55 (9) [0] | 22 (20) [68] | 0 (8) [36] | - | - | - | - |

**Table S1: Number of cells observed for division, death and lost events in each generation.** For each condition of $CD8^+$ T cell with 1U, 3U and 10U IL-2, measurements from two independent but identical experiment setup are pooled. The total number of cells in each generation (i.e. divided + dead + lost cells) is equal to twice of divided cells from previous generation. A cell is considered lost if it becomes indistinguishable to the nearby cells, or survives until the end of given experiment time frame.

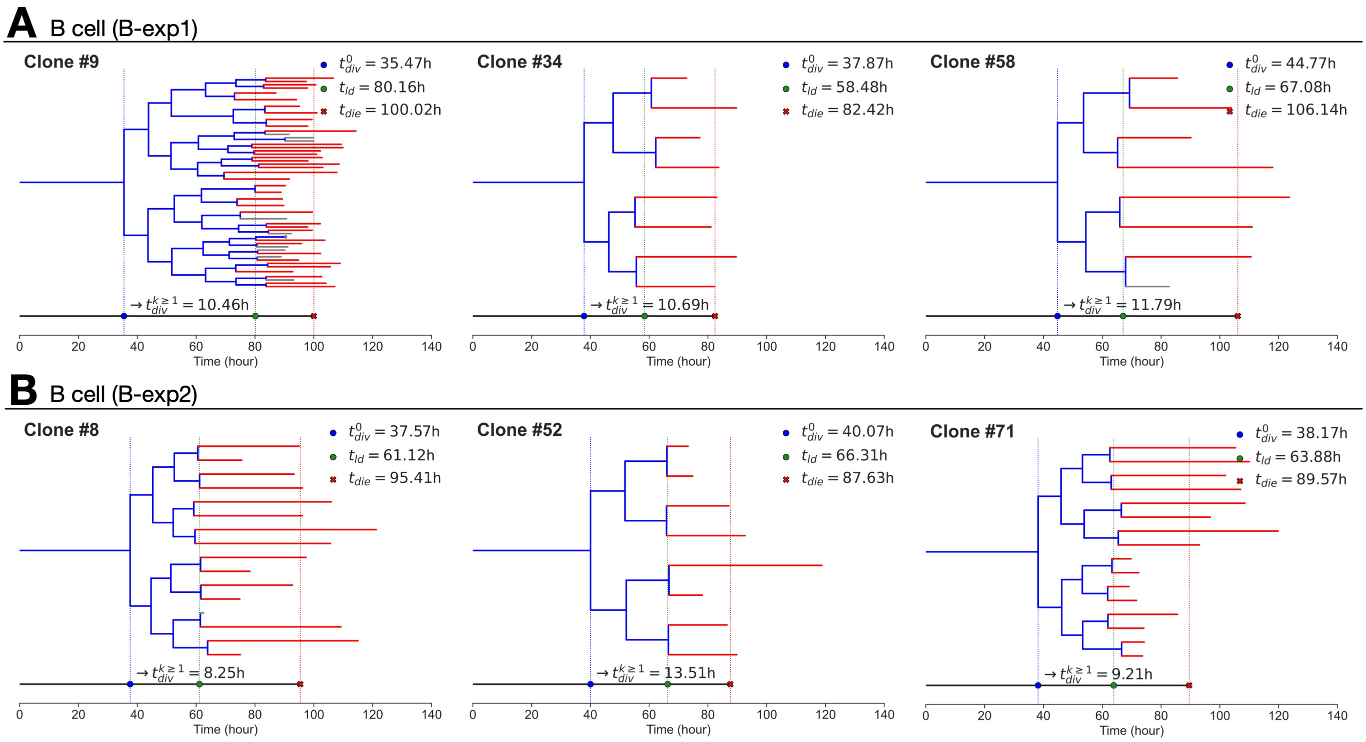


**Figure S1: Example family and clonally collapsed trees of B cells in the filming datasets.** Each panel shows one family. A horizontal line represents the lifetime of a cell in dividing (——), dying (——), or lost (——) state. Clonally collapsed tree is shown as a single time-line (——) below the family tree. Time to first division (●), average time to last division (●), average time to death (×) and average subsequent division time ($t^{k\geq1}_{div}$) are annotated on the collapsed line. These values are shown in the legend. (**A**) Experiment CpG-stimulated B cells (B-exp1). (**B**) Repeat of B cell experiment (B-exp2).

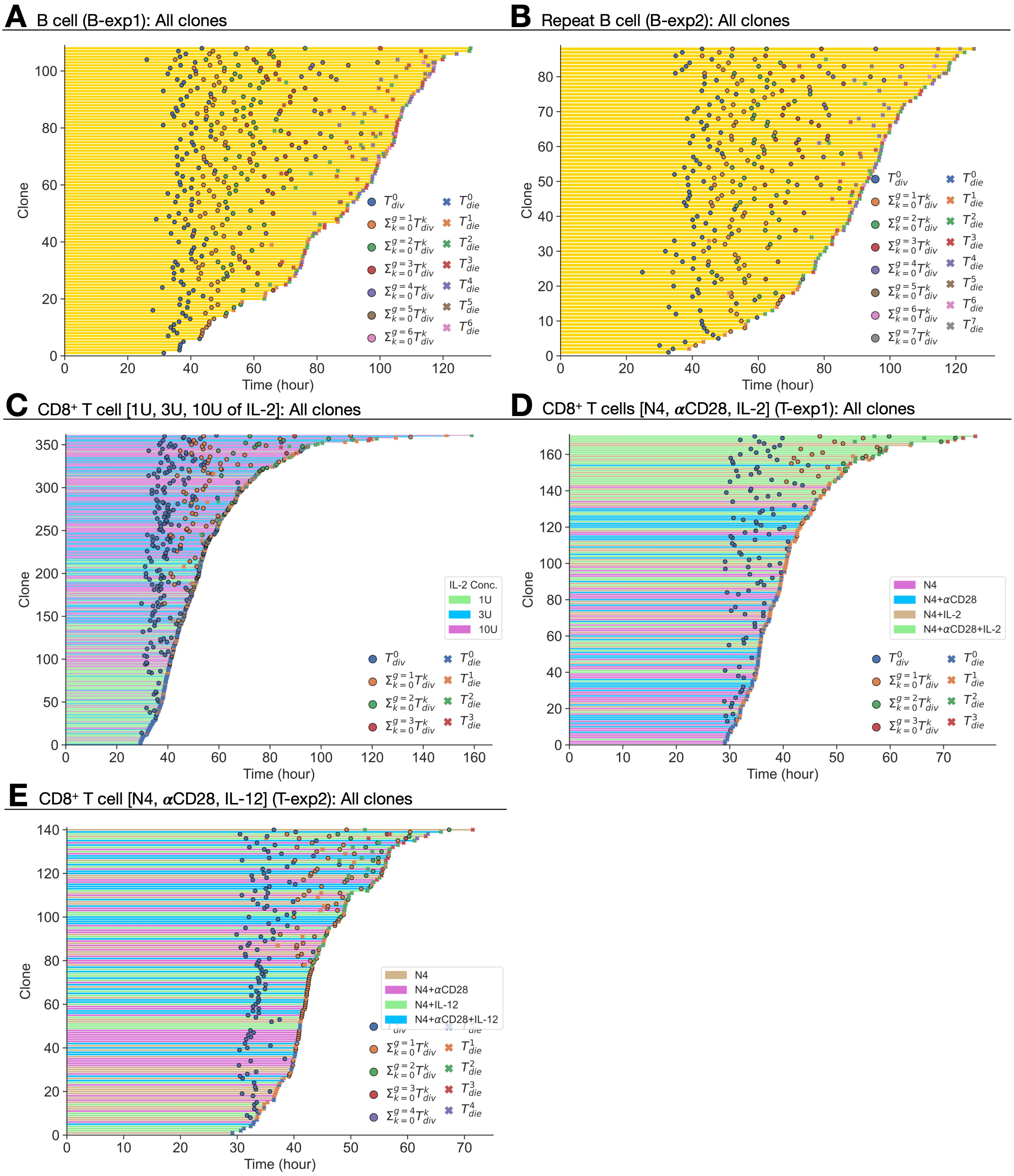


**Figure S2: Clonally collapsed clones for all founder B and CD8$^+$ T cells.** For CD8$^+$ T cell data with 1U, 3U and 10U of IL-2, measurements from two independent but identical experiment setup are aggregated. **(A-F)** Clonally collapsed trees for all families before the filtering. The average division (•) and death (×) times are marked per family, where the colors represent generation that the event was observed. The lost times are not shown.

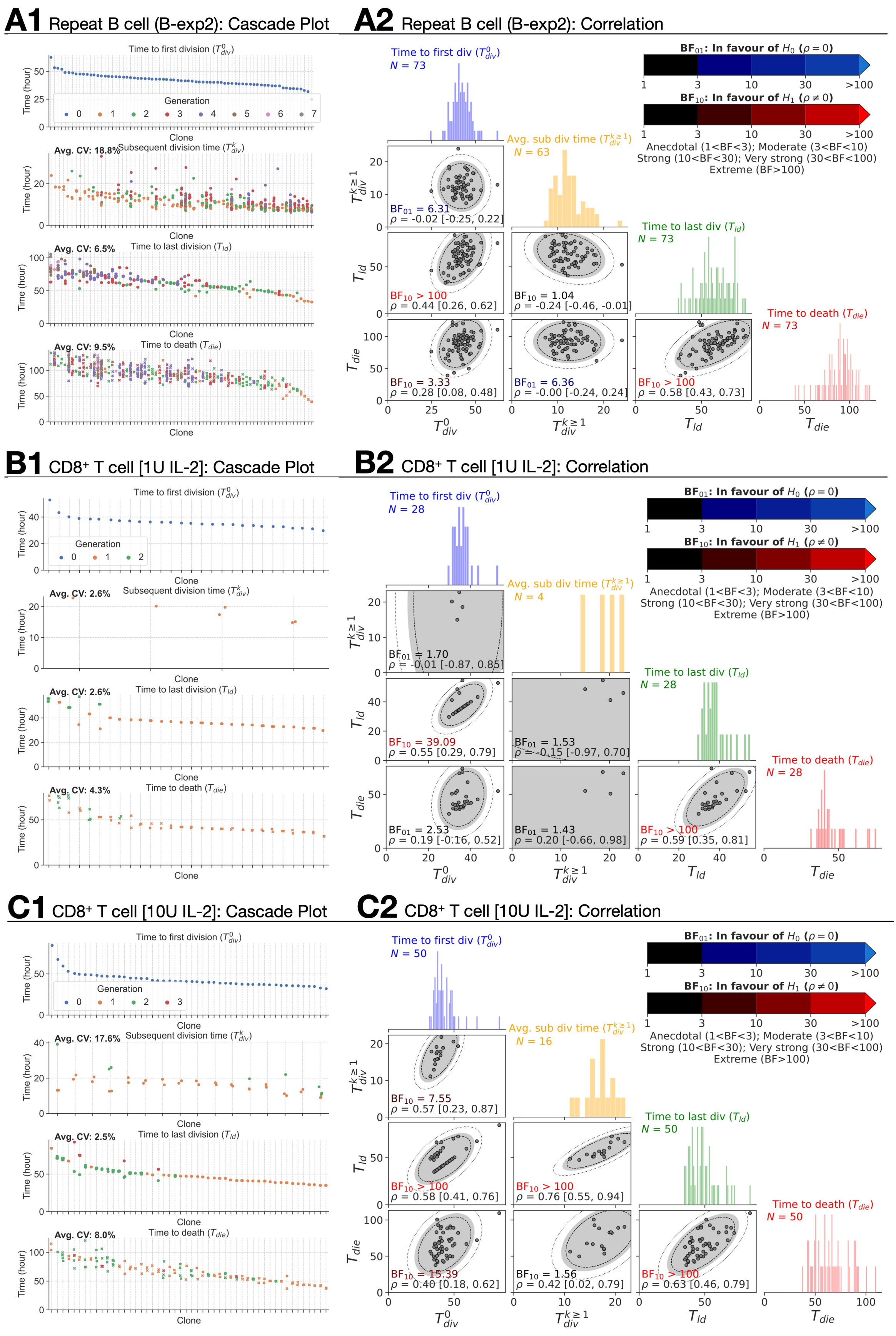


**Figure S3: Extracting times to fates from the repeat of CpG-stimulated B cells and CD8$^+$ T cells in the presence of 1U or 10U IL-2.** For CD8$^+$ T cell data, measurements from two independent but identical experiment setup are aggregated. **(A1,B1,C1)** Four key Cyton2 variables, $T_{div}^0$, $\{T_{div}^k\}_{k\geq1}$, $T_{ld}$ (replacing $T_{dd}$ as a proxy measure) and $T_{die}$ for all cells in each family are shown as a series of cascade plots. Average coefficient of variation (CV) for each variable is annotated. The lost cells are not shown. **(A2,B2,C2)** Pair-plot of the Cyton2 variables. Each point represents a family consists of $T_{div}^0$, average $\{T_{div}^k\}_{k\geq1}$, average $T_{ld}$ and average $T_{die}$. Distributions of the times are collated into 1 hour time interval and shown in the diagonal panels. Correlation coefficient ($\rho$) was estimated using bivariate normal distribution with 95% credible interval for each pair. 90%, 95% and 99% density regions are plotted over the data. Given two hypotheses ($H_0$: $\rho = 0$ and $H_1$: $\rho \neq 0$), Bayes Factor ($BF_{01} = 1/BF_{10}$) was calculated. If the data is more probable under $H_0$, then it is $BF_{01}$ times more favoured than $H_1$ (*blue-scale*), otherwise $H_1$ is $BF_{10}$ (*red-scale*) times more favoured than $H_0$.

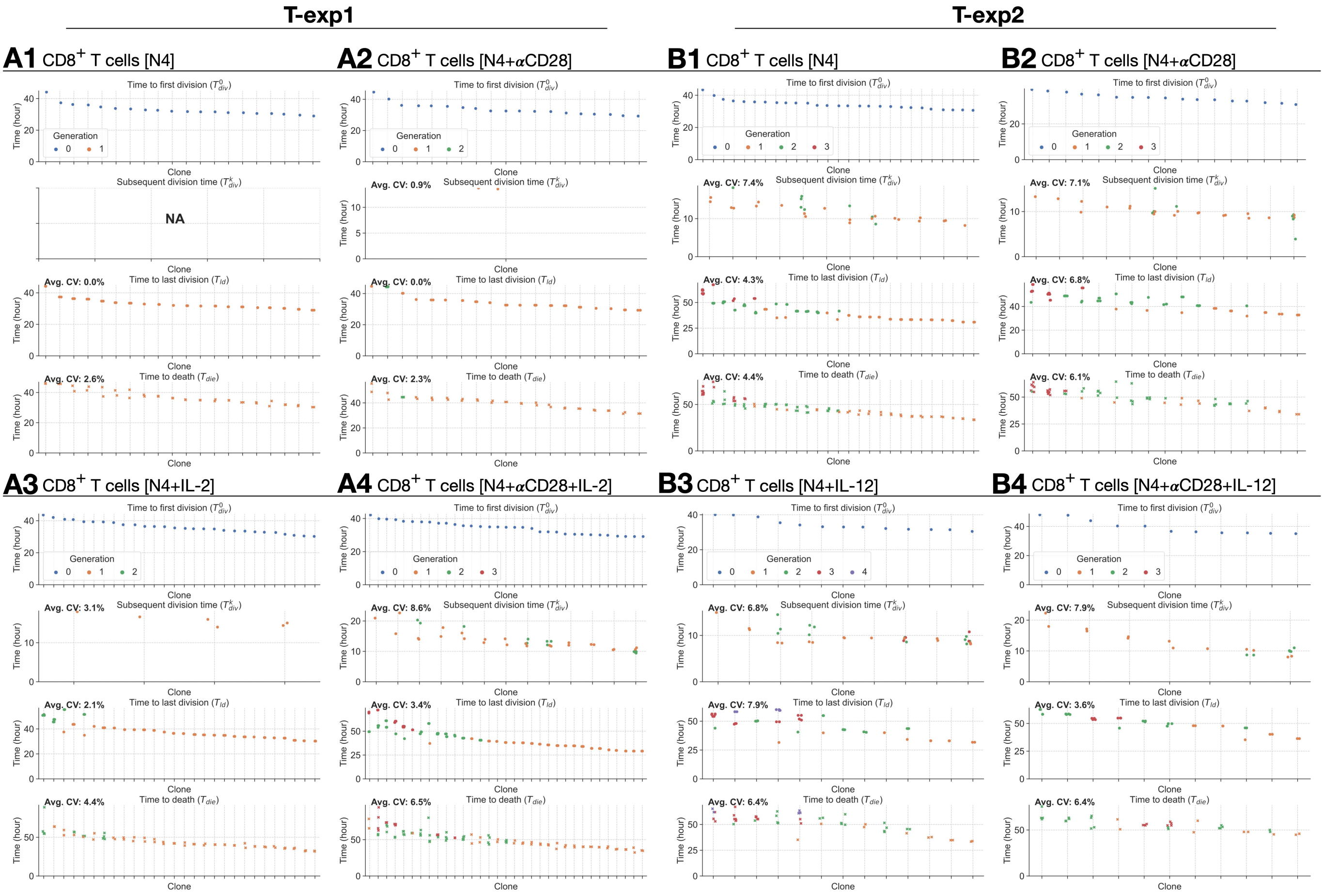


**Figure S4: Extracting times to fates from CD8$^+$ T cell costimulation experiments.** Four key Cyton2 variables, $T^0_{div}$, $\{T^k_{div}\}_{k\geq1}$, $T_{ld}$ (replacing $T_{dd}$ as a proxy measure) and $T_{die}$ for all cells in each family are shown as a series of cascade plots. Average coefficient of variation (CV) for each variable is annotated. The lost cells are not shown. **(A1-4)** Experiment T-exp1 consists of N4 only, N4 + $\alpha$CD28, N4 + IL-2 and N4 + $\alpha$CD28 + IL-2. **(B1-4)** Experiment T-exp2 constists of N4 only, N4 + $\alpha$CD28, N4 + IL-12 and N4 + $\alpha$CD28 + IL-12.

| Cell Type | Stimulation | Number of clones ($N$) & Bayes Factor (BF$_{01} = 1/\mathrm{BF}_{10}$) & Correlation Coefficient ($\rho$ [CI]) | | | | | |
|---|---|---|---|---|---|---|---|
| | | $(T^0_{div}, T^{k\geq1}_{div})$ | $(T^0_{div}, T_{ld})$ | $(T^0_{div}, T_{die})$ | $(T^{k\geq1}_{div}, T_{ld})$ | $(T^{k\geq1}_{div}, T_{die})$ | $(T_{ld}, T_{die})$ |
| CD8$^+$ T (T-exp1) | N4 | $N < 2$<br>NA | $N = 20$<br>BF$_{10} > 100$<br>1.00 [1.00, 1.00] | $N = 20$<br>BF$_{10} = 8.31$<br>0.53 [0.22, 0.82] | $N < 2$<br>NA | $N < 2$<br>NA | $N = 20$<br>BF$_{10} = 8.31$<br>0.53 [0.22, 0.82] |
| | N4 + $\alpha$CD28 | $N < 2$<br>NA | $N = 19$<br>BF$_{10} = 85.63$<br>0.67 [0.42, 0.89] | $N = 19$<br>BF$_{10} = 18.83$<br>0.59 [0.30, 0.86] | $N < 2$<br>NA | $N < 2$<br>NA | $N = 19$<br>BF$_{10} = 60.52$<br>0.66 [0.40, 0.89] |
| | N4 + IL-2 | $N = 4$<br>BF$_{01} = 1.30$<br>0.24 [-0.64, 0.99] | $N = 28$<br>BF$_{10} = 29.98$<br>0.54 [0.28, 0.78] | $N = 28$<br>BF$_{10} = 2.09$<br>0.38 [0.06, 0.67] | $N = 4$<br>BF$_{01} = 1.31$<br>-0.24 [-0.99, 0.63] | $N = 4$<br>BF$_{01} = 1.58$<br>-0.12 [-0.96, 0.73] | $N = 28$<br>BF$_{10} > 100$<br>0.69 [0.49, 0.87] |
| | N4 + $\alpha$CD28 + IL-2 | $N = 13$<br>BF$_{10} = 4.17$<br>0.55 [0.17, 0.89] | $N = 33$<br>BF$_{10} = 1.03$<br>0.30 [-0.01, 0.59] | $N = 13$<br>BF$_{01} = 3.28$<br>0.14 [-0.18, 0.45] | $N = 13$<br>BF$_{01} = 1.26$<br>0.33 [-0.14, 0.78] | $N = 33$<br>BF$_{01} = 1.77$<br>0.26 [-0.23, 0.72] | $N = 33$<br>BF$_{10} > 100$<br>0.71 [0.54, 0.87] |
| CD8$^+$ T (T-exp2) | N4 | $N = 12$<br>BF$_{10} = 15.12$<br>0.68 [0.35, 0.94] | $N = 27$<br>BF$_{01} = 2.04$<br>0.22 [-0.13, 0.56] | $N = 27$<br>BF$_{01} = 2.31$<br>0.20 [-0.15, 0.54] | $N = 12$<br>BF$_{10} = 2.69$<br>0.52 [0.10, 0.89] | $N = 12$<br>BF$_{10} = 3.53$<br>0.55 [0.15, 0.90] | $N = 27$<br>BF$_{10} > 100$<br>0.95 [0.91, 0.98] |
| | N4 + $\alpha$CD28 | $N = 12$<br>BF$_{01} = 1.34$<br>0.33 [-0.18, 0.78] | $N = 17$<br>BF$_{01} = 2.84$<br>0.13 [-0.32, 0.57] | $N = 17$<br>BF$_{01} = 2.75$<br>0.15 [-0.30, 0.59] | $N = 12$<br>BF$_{01} = 2.82$<br>-0.02 [-0.55, 0.52] | $N = 12$<br>BF$_{01} = 1.48$<br>0.30 [-0.19, 0.78] | $N = 17$<br>BF$_{10} > 100$<br>0.83 [0.67, 0.96] |
| | N4 + IL-12 | $N = 9$<br>BF$_{10} = 14.97$<br>0.74 [0.41, 0.98] | $N = 13$<br>BF$_{01} = 2.58$<br>0.13 [-0.38, 0.63] | $N = 13$<br>BF$_{01} = 2.39$<br>0.17 [-0.34, 0.66] | $N = 9$<br>BF$_{01} = 2.21$<br>0.14 [-0.48, 0.72] | $N = 9$<br>BF$_{01} = 2.24$<br>0.13 [-0.48, 0.73] | $N = 13$<br>BF$_{10} > 100$<br>0.95 [0.88, 0.99] |
| | N4 + $\alpha$CD28 + IL-12 | $N = 7$<br>BF$_{01} = 1.33$<br>0.32 [-0.33, 0.90] | $N = 11$<br>BF$_{01} = 2.47$<br>0.12 [-0.43, 0.66] | $N = 11$<br>BF$_{01} = 2.48$<br>0.12 [-0.44, 0.66] | $N = 7$<br>BF$_{01} = 1.00$<br>0.40 [-0.23, 0.93] | $N = 7$<br>BF$_{10} = 1.16$<br>0.44 [-0.17, 0.95] | $N = 11$<br>BF$_{10} = 29.19$<br>0.74 [0.45, 0.96] |

BF interpretation: Anecdotal ($1 <$ BF $\leq 3$); Moderate ($3 <$ BF $\leq 10$); Strong ($10 <$ BF $\leq 30$); Very strong ($30 <$ BF $\leq 100$); Extreme (BF $> 100$)

**Table S2: Bayesian independence test of the times to fates extracted from CD8$^+$ T cell costimulation filming datasets.** For each pair of the times to fates, the correlation coefficient was estimated with 95% credible interval using bivariate normal distribution and Bayes Factor (BF) was calculated. Given two hypotheses (H$_0$: $\rho = 0$ and H$_1$: $\rho \neq 0$), if the data is more probable under H$_0$, then it is BF$_{01}$ (*blue-scale*) times more favoured than H$_1$, otherwise H$_1$ is BF$_{10}$ (*red-scale*) times more favoured than H$_0$.

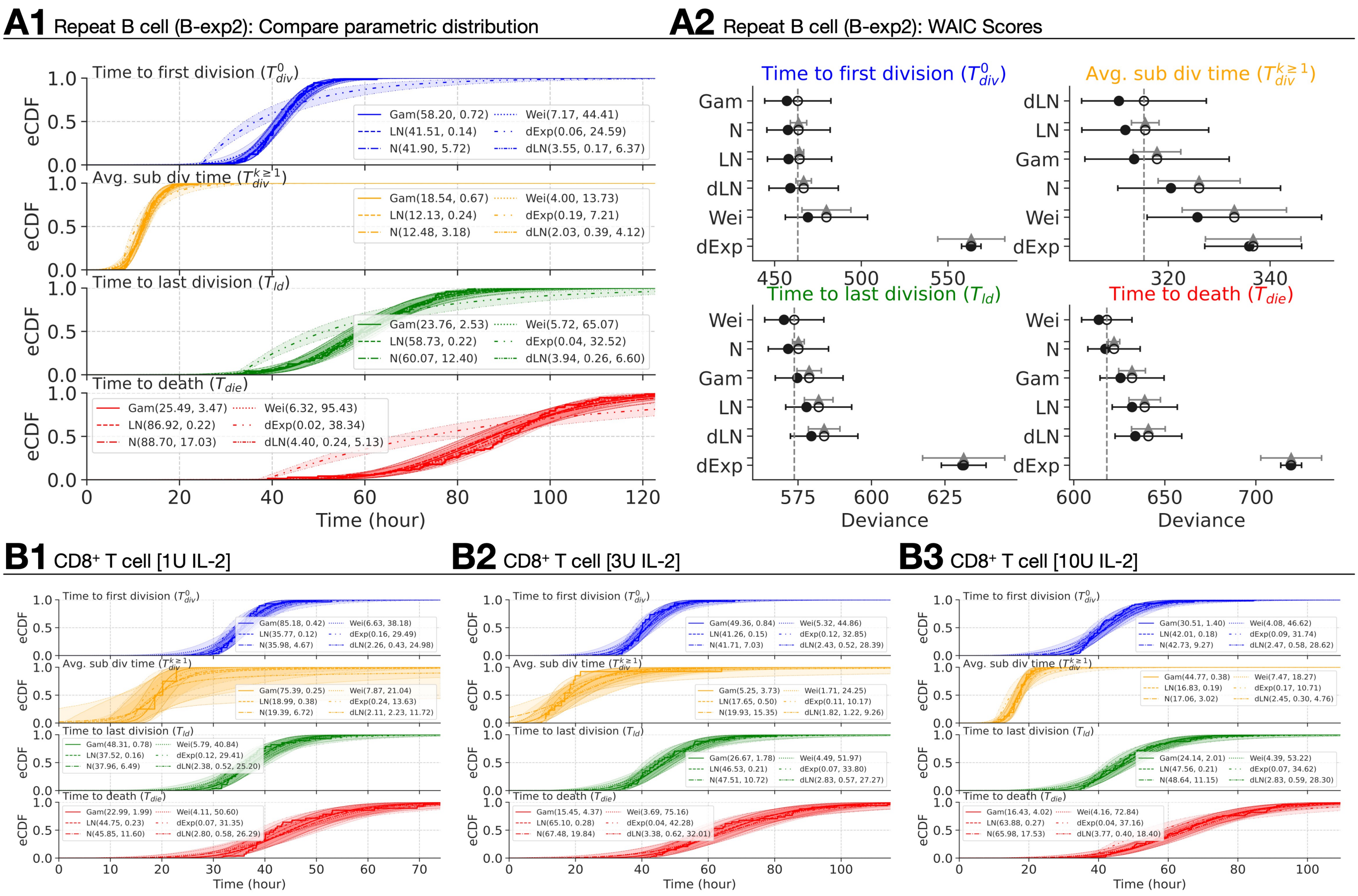


**Figure S5: Best parametric distribution classes.** The eCDFs of measurements of times to fates are presented. Six candidate distribution classes are parameterised by: ($\alpha_G$, $\beta_G$) for Gamma; ($m$, $s$) median and scale for lognormal; ($\mu$, $\sigma$) mean and standard deviation for Normal; ($\alpha_W$, $\beta_W$) for Weibull; ($\lambda$, $c$) rate and shift for delayed Exponential; and, ($m_d$, $s_d$, $c$) median, scale and shift for delayed Lognormal. The CDF of the candidates was plotted by taking mean values of posterior distribution of hyper-parameters. The confidence band was plotted by sampling hyper-parameter values from posterior distribution. **(A1-2)** Repeat of CpG-stimulated B cell (B-cpg2). Rank-ordered WAIC plot is shown. **(B1-3)** CD8$^+$ T cell in the presence of 1U, 3U and 10U of IL-2. For T cell data, measurements from two independent but identical experiment setup are aggregated. Corresponding WAIC plot is shown in **Figure 4B1-3**.

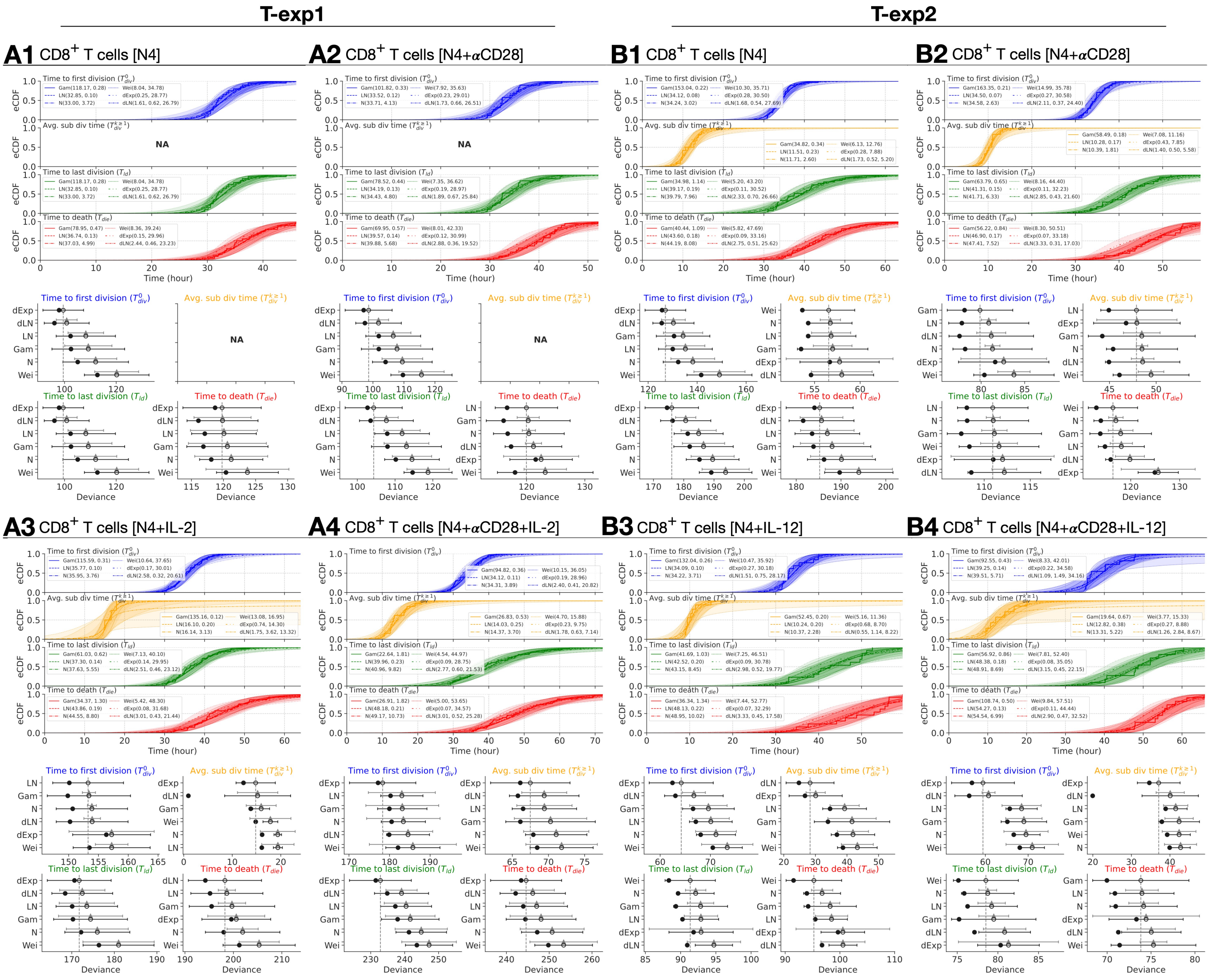


**Figure S6: Best parametric distribution classes.** The eCDFs of measurements of times to fates are presented. Four candidate distribution classes are parameterised by: ($\alpha_G$, $\beta_G$) for Gamma; ($m$, $s$) median and scale for lognormal; ($\mu$, $\sigma$) mean and standard deviation for Normal; ($\alpha_W$, $\beta_W$) for Weibull; ($\lambda$, $c$) rate and shift for delayed Exponential; and, ($m_d$, $s_d$, $c$) median, scale and shift for delayed Lognormal. The CDF of the candidates was plotted by taking mean values of posterior distribution of hyper-parameters. The confidence band was plotted by sampling hyper-parameter values from posterior distribution. **(A1-4)** Experiment T-exp1 consists of N4 only, N4 + $\alpha$CD28, N4 + IL2 and N4 + $\alpha$CD28 + IL-2. **(B1-4)** Experiment T-exp2 constists of N4 only, N4 + $\alpha$CD28, N4 + IL-12 and N4 + $\alpha$CD28 + IL-12. Minimum of two observations per variable was required for the calculation.

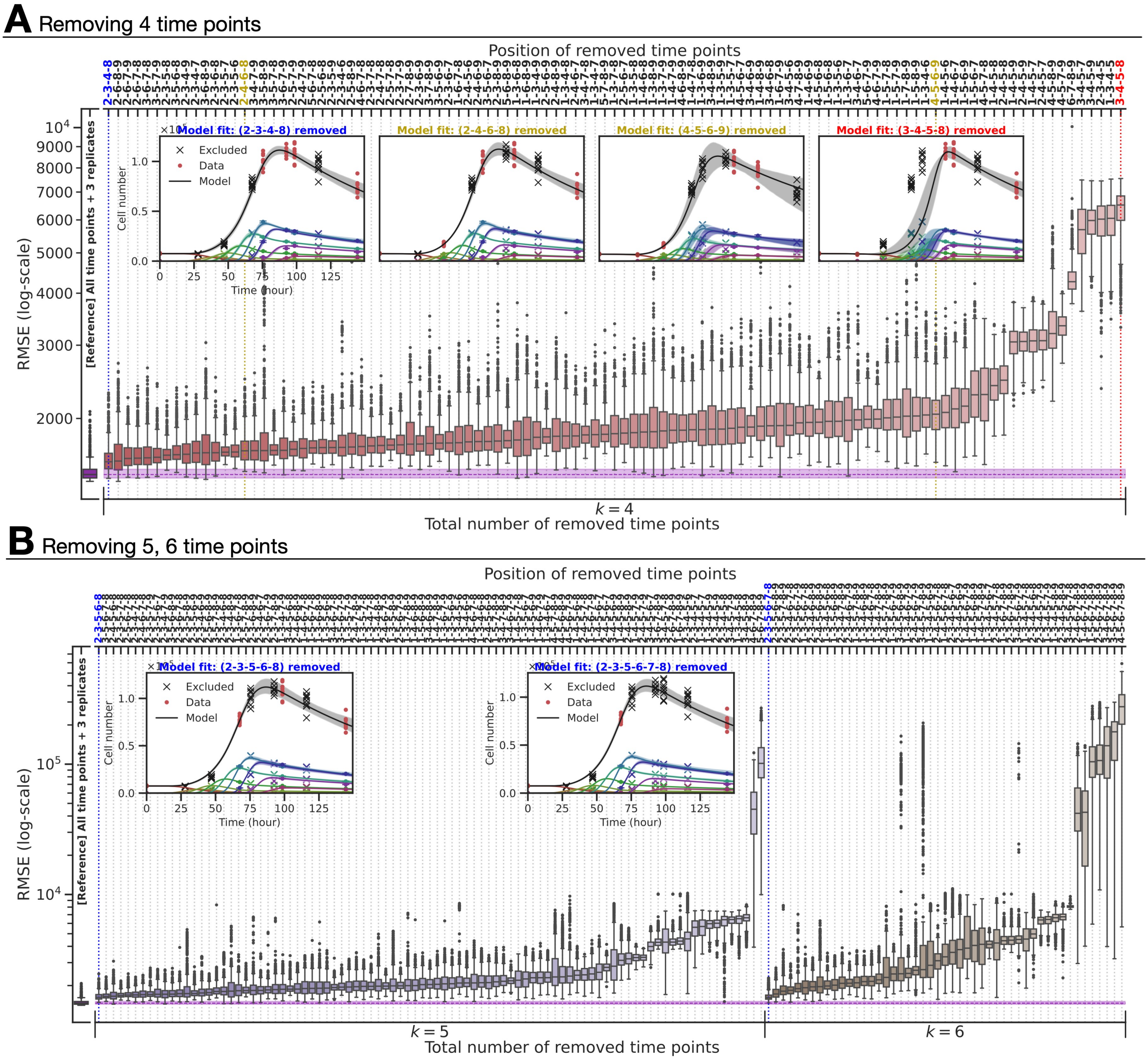


**Figure S7: The accuracy of the model fit with CpG-stimulated Bim$^{-/-}$ B cell FACS data.** The root-mean-squared error (RMSE) was evaluated over all available data points after fitting the Cyton2 model to the synthetic datasets. Similarly, the reference RMSE (*purple*) was obtained after fitting the model to datasets assuming only three replicates are available while maintaining all time points. **(A)** All possible combinations of positions of time points for $k = 4$ case. The best (*blue*) and worst (*red*) examples of Cyton fits are shown. **(B)** Best examples for $k = 5, 6$ are shown. The worst cases failed to provide good fits (not shown), resulting in large confidence bands and an order of magnitude difference in RMSE.

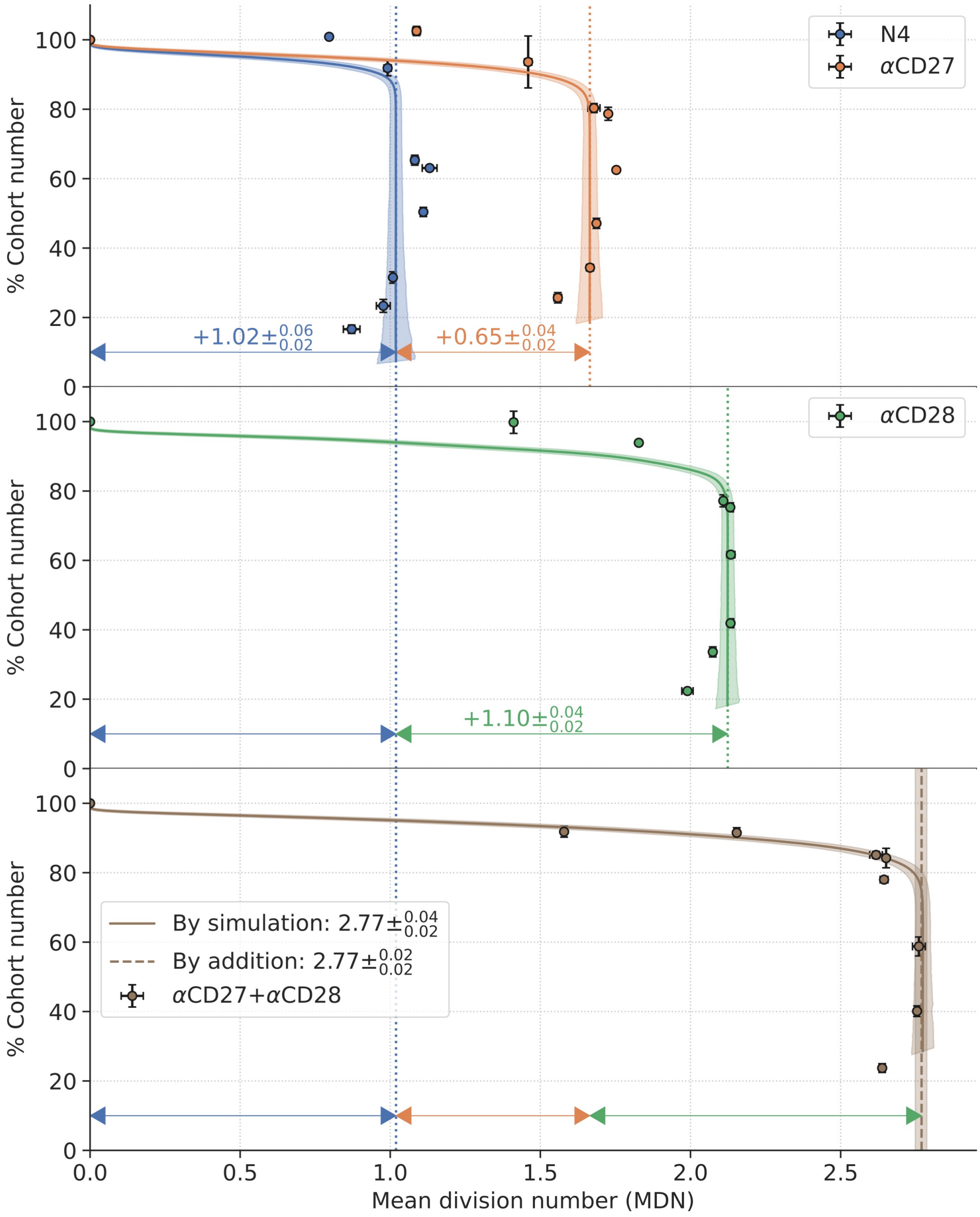


**Figure S8: Linear sum of the signals from simulated trees (correspond to Fig.3 in Marchingo et al. 2014).** For each harvested time points from FACS data, percentage cohort number (with respect to the first time point) as a function of MDN is shown (•: mean ± SEM). The Agent-Based Model was used to generate family trees. The times to first, to destiny and to death were randomly sampled from the estimated *normal* distributions by fitting the reduced Cyton2 model to N4, $\alpha$CD27 and $\alpha$CD28 datasets simultaneously. Note that the subsequent division time was set as a constant to be consistent with the model. To match the data, each round of the simulation was initialised with 6066 (N4), 8252 ($\alpha$CD27) and 8377 ($\alpha$CD28) clones and ran for $t \in [0, 140]$ with $\Delta t = 0.5$ in hours. This process was repeated 1000 times to obtain 95% confidence bands around the mean. Increase in MDN is labelled in each panel with arrows. The predicted MDNs for $\alpha$CD27+$\alpha$CD28 were calculated either by summing the contribution from each individual stimulation (- - -) or simulating the trees directly with the summed timers (——).

## References


1. Marchingo, Julia M et al. (2014). "Antigen affinity, costimulation, and cytokine inputs sum linearly to amplify T cell expansion". *Science* 346, pp. 1123–1127.
